# Single-cell profiling reveals a shared proinflammatory macrophage signature across multiple organs in myopia

**DOI:** 10.1038/s41421-025-00835-8

**Published:** 2025-12-02

**Authors:** Jiaqi Meng, Ye Zhang, Mengchao Zhu, Yu Du, Yunqian Yao, Shuyu Liu, Wenwen He, Xiangjia Zhu

**Affiliations:** 1 Department of Ophthalmology, Eye & ENT Hospital, Fudan University, Shanghai, China; 2 Key laboratory of Myopia and Related Eye Diseases, NHC; Key laboratory of Myopia and Related Eye Diseases, Chinese Academy of Medical Sciences, Shanghai, China; 3 Key Laboratory of Medical Neurobiology, Fudan University, Shanghai, China

**Keywords:** Innate immunity, Mechanisms of disease, Gene expression profiling, Reprogramming

## Abstract

Myopia is a leading cause of visual impairment, with its prevalence rising rapidly worldwide. Our prior investigations suggest that cross-organ communication, involving the eye, brain, and gut, may play a role in myopia. However, the extent of this cross-organ communication in myopia remains unclear. To elucidate the underlying mechanisms, this study generates a comprehensive pan-tissue transcriptome profile of myopic mice covering eye, brain, blood, bone marrow, spleen, thymus, intestines, liver, kidney, lung, and adrenal gland using single-cell RNA sequencing (scRNA-seq). Widespread immunologic alterations in myopia are identified, characterized by a significant increase in macrophage abundance and macrophage-mediated cell communications across multiple tissues. Notably, these macrophages exhibit a cross-tissue proinflammatory phenotype, which is marked by significant activation of the hypoxia pathway, with upregulation of key markers, including Car1, HIF-1α, and reactive oxygen species, a pattern also observed in the blood of myopic patients. Further analysis suggested that hypoxia stress likely regulates the energy metabolism of proinflammatory macrophages. Inhibition of the hypoxia pathway suppressed the proinflammatory phenotype of macrophages and their hypoxia-related gene expression in myopic mice, reducing the degree of myopia. More importantly, analysis of a large cohort of 114,661 patients reveals 16 extraocular diseases with a myopia-biased prevalence. Our findings underscore the link between myopia and extraocular diseases and suggest that proinflammatory macrophages may potentially serve as the shared mechanism across organs.

## Introduction

Myopia has emerged as a predominant cause of global visual impairments^1^, contributing to a range of sight-threatening complications affecting various ocular tissues, including lens, retina, and optic nerve^2–4^. Unfortunately, effective interventions are hindered by the unclear pathogenesis of this condition.

It has long been acknowledged that myopia simply results from abnormal refractive development. However, recent research has unveiled an inflammatory intraocular microenvironment in myopic eyes, characterized by elevated levels of proinflammatory cytokines (CCL2, TNF-α, IL-6, and IFN-γ) in tear fluid and aqueous humor^5,6^, along with heightened inflammatory infiltrates in the retina and sclera^7,8^. Furthermore, our latest work has established an association between myopia and anxiety, implicating eye–brain communication and compromised blood–tissue barriers due to increased monocyte infiltration as underlying mechanisms^9^. The discovery of gut microbiota dysbiosis in myopic patients also suggests the crosstalk between the myopic eye and the intestine^10^. Other research suggested that interventions, such as dietary supplements involving omega-3, crocetin, and lactoferrin, have emerged as a significant area of research in myopia prevention and treatment^11–15^. Thus, we hypothesize that myopia may involve pathological processes beyond the ocular tissues, and that its systemic impacts may be associated with an inflammatory mechanism. However, the link between myopia and diseases of other organs has been rarely studied, let alone the shared underlying mechanisms.

Recent advancements in single-cell RNA sequencing (scRNA-seq) enable a comprehensive assessment of cellular landscapes across organs, facilitating the exploration of complex biological system^16–19^. Utilizing scRNA-seq analysis to uncover cross-tissue similarities may provide valuable insights into the shared mechanisms underlying myopia.

In this study, we applied scRNA-seq to perform integrated profiling of pan-tissue transcriptome covering 15 types of tissues in a myopic mouse model. We found significant immunologic alterations induced by myopia, particularly marked by increased macrophage counts and heightened interaction activity across multiple tissues. Strikingly, macrophages in myopic mice exhibited a cross-tissue proinflammatory phenotype, characterized by upregulation of hypoxia-related genes, including *Car1*, *Hif-1α*, and reactive oxygen species (ROS), which was also recapitulated in human tissues. Further analysis suggested that hypoxia stress likely regulated the energy metabolism of proinflammatory macrophages. Inhibition of the hypoxia pathway could suppress the proinflammatory phenotype of macrophages and their hypoxia-related gene expression in myopic mice, reducing the degree of myopia. Importantly, in a large cohort of 114,661 patients with myopia, our analysis revealed increased prevalence in 16 extraocular diseases. Overall, our findings highlight a potential link between myopia and extraocular organs and suggest the involvement of hypoxia-related proinflammatory macrophages in multiple organs.

## Results

### Myopia induction generates pan-tissue immunologic alterations in mice

**Fig. 1:**
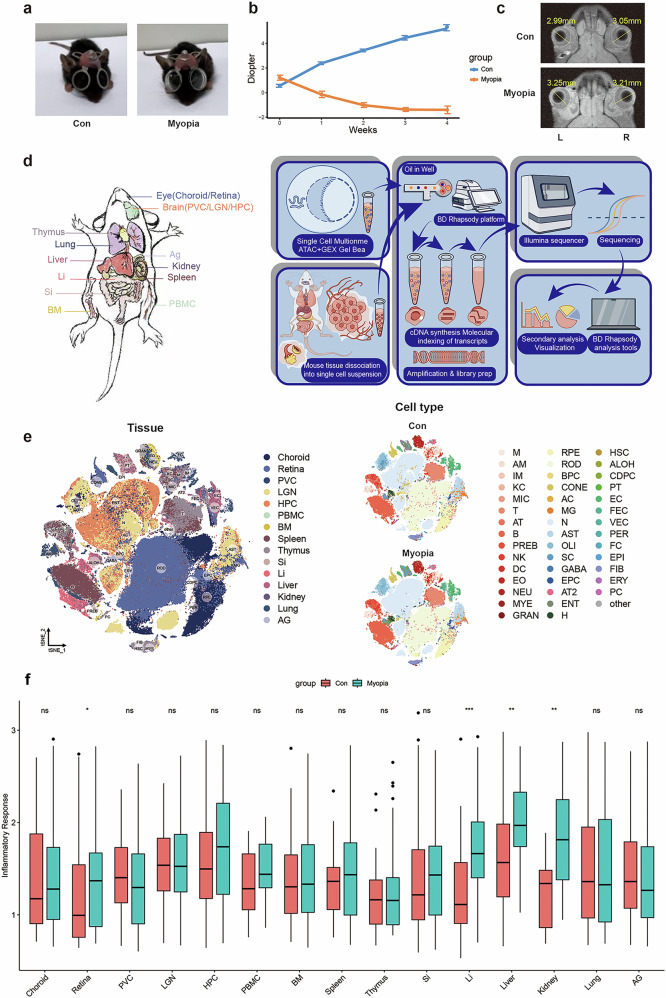
Construction of pan-tissue transcriptome profile of myopic mice by scRNA-seq. **a** Representative photographs contrasting a sham operated mouse (control, Con) with a myopic mouse wearing a –25 D lens to induce myopia in both eyes. **b** Ocular refraction measurements comparing the control and myopia groups (*n* = 20 per group). **c** Sagittal ocular MRI images illustrating significantly elongated AL in the myopia group compared to the control group. Yellow lines indicate AL measurement. **d** Schematic depiction outlining the scRNA-seq methodology. **e** The t-SNE plots visualizing distinct cell types across 15 tissues via scRNA-seq. **f** The box plot visualizing the comparisons of the “inflammatory response” gene set enrichment scores in each tissue between the control and myopia group. PVC primary visual cortex, LGN lateral geniculate nucleus, HPC hippocampus, PBMC peripheral blood mononuclear cell, BM bone marrow, Si small intestine, Li large intestine, AG adrenal gland, M macrophage, AM alveolar macrophage, IM interstitial macrophage, KC Kupffer cell, MIC microglia, T T cell, AT activated T cell, B B cell, PREB pre B cell, NK natural killer cell, DC dendritic cell, EO eosinophil, NEU neutrophil, MYE myeloid cell, GRAN granulocyte, RPE retinal pigment epithelial cell, ROD rod cell, BPC bipolar cell, CONE cone cell, AC amacrine cell, MG muller glia cell, N neuron, AST astrocyte, OLI oligodendrocyte, SC Schwann cell, GABA GABAergic neuron, EPC ependymal cell, AT2 AT2 cell, ENT enterocyte, H hepatocyte, HSC hepatic stellate cell, ALON ascending loop of henle, CDPC collecting duct principal cell, PT proximal tubule cell, EC endothelial cell, FEC fenestrated endothelial cell, VEC vascular endothelial cell, PER pericyte, FC fasciculata cell, EPI epithelial cell, FIB fibroblast, ERY erythroid cell, PC proliferating cell.

To construct a comprehensive transcriptome profile of myopia across multiple tissues at a single-cell resolution, we established a myopic mouse model by attaching a –25 diopter (D) lens over both eyes in mice for 4 weeks, with sham operated mice serving as controls (Con, Fig. 1a). After 4 weeks, the refraction of the eyes in the myopia group was significantly more myopic than those of the control group (Fig. 1b), and axial lengths (ALs) in both eyes of the myopia group were longer than the controls (Fig. 1c), collectively confirming the successful establishment of the myopic mouse model. Then, we isolated 15 tissues from eye (choroid/retina), brain (primary visual cortex (PVC)/lateral geniculate nucleus (LGN)/hippocampus (HPC)), immune tissues (peripheral blood mononuclear cell (PBMC)/bone marrow (BM)/spleen/thymus), and other organs (small intestine/large intestine/liver/ kidney/lung/adrenal gland) and processed them into single-cell suspensions for sequencing (Fig. 1d). Following quality control, a total of 200,127 qualified cells were remained and further earmarked for in-depth analysis. Information of gene types and quantities in each sample was provided in Supplementary Fig. S1.

Based on the expression of classical cell type-specific markers, we totally identified 44 cell types across the 15 tissues (Fig. 1e). Given that our prior studies showed increased inflammatory infiltration in organs including the eye and brain in myopic mice, we further calculated the enrichment score of “inflammatory response” hallmark gene set in each tissue and found the score was consistently upregulated in multiple organs, particularly in retina, large intestine, liver and kidney (Fig. 1f), suggesting that immune cells may play a key role in myopia. Furthermore, compared to control group, myopia group showed a distinct cell distribution especially for immune cell distribution, across multiple tissues (Supplementary Fig. S2). Specifically, there was an increasing tendency of macrophages in myopic mice across various tissues, including the PBMC, BM, spleen, small and large intestines, and liver (Supplementary Figs. S2, S3). These results indicate significant immunologic alterations across multiple tissues induced by myopia.

### Immunologic alterations exhibit consistently increased abundance and altered gene expression patterns of macrophages across most tissues in myopic mice

**Fig. 2:**
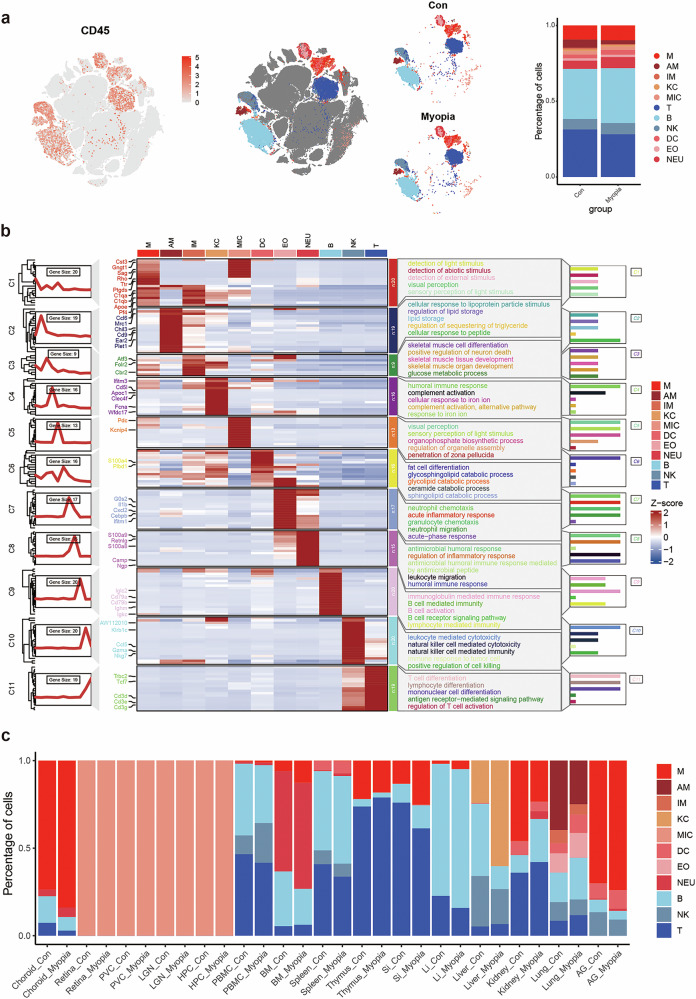
Immunologic alterations exhibit increased macrophages across most tissues in myopic mice. **a** t-SNE plots showing that immune cells accumulated in the tissues of myopic mice. Left, t-SNE plot highlighting the CD45-positive cells. Middle, t-SNE plots of major immune cell types. Right, t-SNE plots and proportions of major immune cell types in the control and myopia groups. **b** Heatmap illustrating distinct expression patterns in different immune cells based on cell type-specific marker genes. **c** Comparison of the percentages of major immune cell types in each tissue between the control and myopia groups. M macrophage, AM alveolar macrophage, IM interstitial macrophage, KC Kupffer cell, MIC microglia, T T cell, B B cell, NK natural killer cell, DC dendritic cell, EO eosinophil, NEU neutrophil. For tissue type: PVC primary visual cortex, LGN lateral geniculate nucleus, HPC hippocampus, PBMC peripheral blood mononuclear cell, BM bone marrow, Si small intestine, Li large intestine, AG adrenal gland.

To further explore the shared immunologic alterations across different tissues in myopic mice, we performed integrative clustering among immune cells (CD45^+^) (Fig. 2a). Thereinto, immune cells were divided into seven major types: macrophages, T cells, B cells, natural killer (NK) cells, dendritic cells (DCs), eosinophils, and neutrophils; and macrophages were further subdivided into monocyte-derived macrophages and resident macrophages including microglia in the retina and brain, Kupffer cells in liver, alveolar and interstitial macrophages in the lung (Fig. 2a). We visualized the distinctive markers of each immune cell type in a heatmap, and found that macrophages and the resident microglia showed an enrichment of genes related with the detection of light stimulus and visual perception (Fig. 2b). Among the immune cells, the percentages of macrophages were significantly elevated in myopic mice across various tissues, including the choroid, PBMC, BM, small intestine, large intestine, and liver (Fig. 2c), which highlights a consistent increase in macrophage abundance regarding the immune cell composition across multiple tissues induced by myopia.

**Fig. 3:**
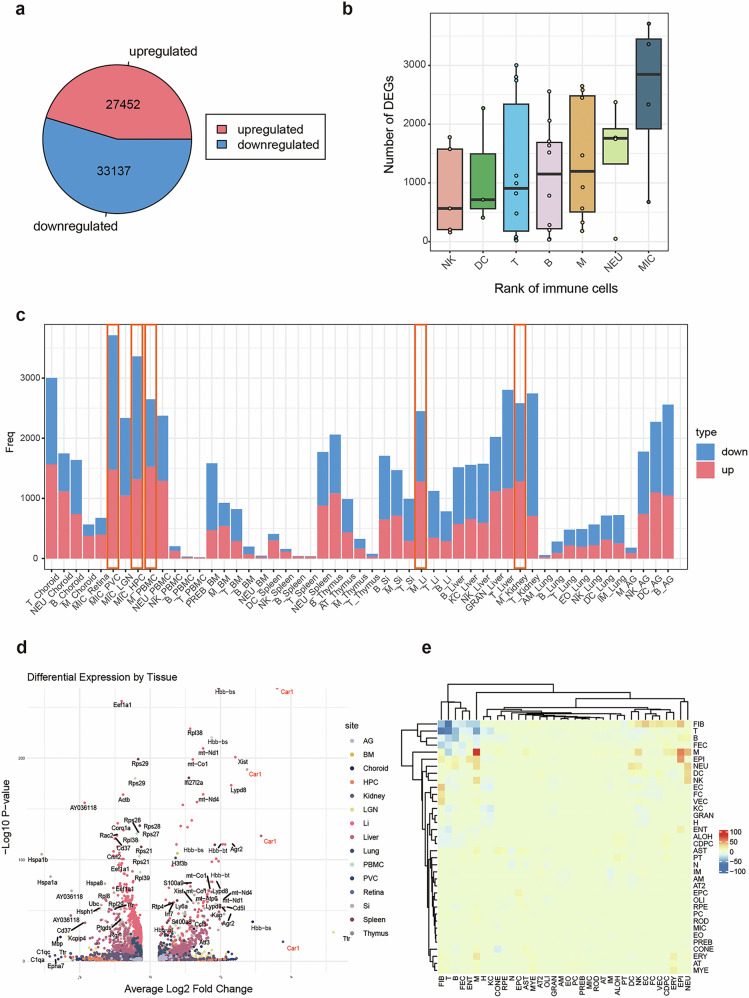
Immunologic alterations exhibit significant multi-tissue gene expression alterations of macrophages across organs in myopic mice. **a** Total number of DEGs in immune cells of myopic mice. **b** Ranking of the number of DEGs among immune cell types in myopic mice. **c** Frequency distribution of DEGs among different immune cell types across tissues of myopic mice. Orange boxes indicate the high frequencies of DEGs in macrophages and resident microglia in multiple tissues of myopic mice. **d** Volcano plot showing the distribution of DEGs across tissues of myopic mice compared to controls. **e** Heatmap showing the highest ligand–receptor interaction activity identified in macrophages of myopic mice. PVC primary visual cortex, LGN lateral geniculate nucleus, HPC hippocampus, PBMC peripheral blood mononuclear cell, BM bone marrow, Si small intestine, Li large intestine, AG adrenal gland. For cell type: M macrophage, AM alveolar macrophage, IM interstitial macrophage, KC Kupffer cell, MIC microglia, T T cell, AT activated T cell, B B cell, PREB pre B cell, NK natural killer cell, DC dendritic cell, EO eosinophil, NEU neutrophil, MYE myeloid cell, GRAN granulocyte, RPE retinal pigment epithelial cell, ROD rod cell, CONE cone cell, N neuron, AST astrocyte, OLI oligodendrocyte, AT2 AT2 cell, ENT enterocyte, H hepatocyte, ALON ascending loop of henle, CDPC collecting duct principal cell, PT proximal tubule cell, EC endothelial cell, FEC fenestrated endothelial cell, VEC vascular endothelial cell, FC fasciculata cell, EPI epithelial cell, FIB fibroblast, ERY erythroid cell, PC proliferating cells.

Then, we further investigated gene expression patterns of macrophages. Analysis of differentially expressed genes (DEGs) in immune cells revealed a total of 27,452 upregulated and 33,137 downregulated DEGs, of which 40.6% (11,153/27,452) and 37.5% (12,411/33,137) were from macrophages, respectively (Fig. 3a). Among the immune cells, macrophages exhibited the highest number of DEGs, including the microglia, the resident macrophages in the retina and brain, and monocyte-derived macrophages (Fig. 3b). Besides, microglia displayed notably high frequencies of DEGs in the PVC and HPC, and macrophages displayed notably high frequencies of DEGs in PBMC, large intestine and kidney of myopic mice (Fig. 3c). Among these immune-related DEGs, the most upregulated gene, *Car1*, which encodes a zinc-metalloenzyme carbonic anhydrase associated with hypoxia-related regulation of macrophage activity, was shared across intestines, liver and kidney (Fig. 3d). Additionally, cell communication pattern also showed a significantly higher activity of ligand–receptor interaction among macrophages, as well as between the macrophages and epithelial cells, in the myopia group, compared to the controls (Fig. 3e).

Overall, the immunologic alterations revealed a significant increase in both the abundance and alterations in gene expression patterns of macrophages, suggesting the pivotal role of macrophages in the systemic impacts across multiple tissues in myopic mice.

### Proinflammatory macrophage phenotype is shared across multiple tissues in myopic mice

**Fig. 4:**
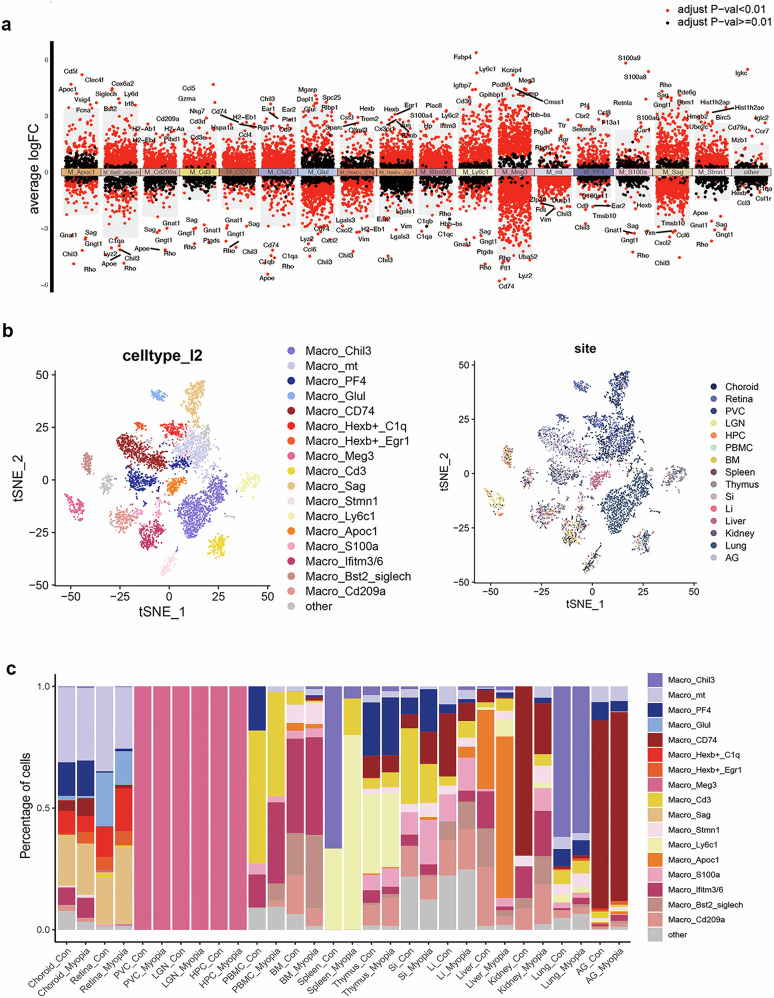
Proinflammatory macrophage phenotype is shared across multiple tissues in myopic mice. **a** Volcano plots highlighting distinctive markers of 17 macrophage subsets. **b** t-SNE plots displaying 17 macrophage subsets and their distributions in different tissues. **c** Percentages of each macrophage subset in the control and myopia groups, with comparisons across tissues. Compared to controls, the myopia group showed significantly increased proinflammatory subsets (Macro_Apoc1, Macro_S100a, Macro_Ifitm3/6, etc.) and decreased anti-inflammatory subsets (Macro_Chil3, Macro_Glul, etc.). PVC primary visual cortex, LGN lateral geniculate nucleus, HPC hippocampus, PBMC peripheral blood mononuclear cell, BM bone marrow, Si small intestine, Li large intestine, AG adrenal gland.

To elucidate the specific phenotype of macrophages in myopic mice, we further categorized 17 subsets of macrophages and visualized the distinct gene markers of each subset in volcano plots (Fig. 4a)^20–23^. The distribution of each subset of macrophages in different organs is presented in Fig. 4b. Compared to the control group, the myopia group showed significantly increased proinflammatory subsets (Macro_Apoc1, Macro_S100a, etc.) and decreased anti-inflammatory subsets (Macro_Chil3, Macro_Glul, etc). In particular, in the tissues with higher inflammatory response enrichment scores in the myopia group, a consistent increase in proinflammatory subsets of macrophages but a decrease in anti-inflammatory subsets were observed. Retina macrophages showed an increase in the proinflammatory subset Macro_Sag and a decrease in the subset Macro_Glul. Macrophages in the myopia group upregulated proinflammatory *Ifitm3/6*, *Apoc1*, and *Bst2-Siglech* in the large intestine, proinflammatory *ly6c1*, *Apoc1*, and *S100a* in the liver, and *ly6c1* and *S100a* in the kidney (Fig. 4c).

Thus, in addition to the increased abundance and altered gene expression patterns of macrophages in myopic mice, we further clarified that the macrophages induced by myopia predominantly exhibited a pro-inflammatory phenotype across multiple tissues.

### The proinflammatory macrophages in myopic mice are characterized by activation of hypoxia pathway

**Fig. 5:**
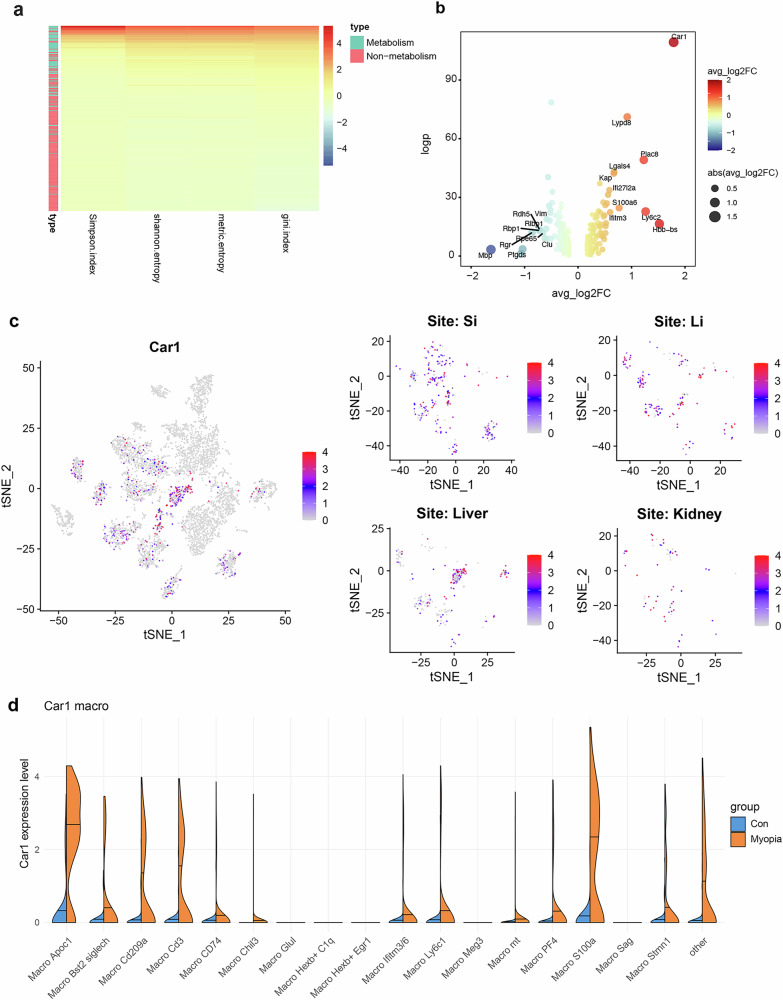
The proinflammatory macrophages in myopic mice reveal a connection to the hypoxia pathway. **a** Heatmap showing variance in metabolism and non-metabolism pathways in macrophages of myopic mice compared to controls. **b** Volcano plot displaying macrophage DEGs in myopic mice, with *Car1* showing the highest upregulation. **c** The t-SNE plot showing the Car1-positive macrophages and their distribution in representative tissues including small and large intestines, liver and kidney. **d** The comparisons of *Car1* expression in different macrophage subsets between the control and myopia groups showing that *Car1* was predominantly upregulated among the proinflammatory subsets of macrophages.

To unveil the molecular pathway that may contribute to the above proinflammatory phenotype of macrophages, we assessed regulatory pathways and genes in the macrophages of myopic mice. Interestingly, these macrophages exhibited higher variance in metabolism-related pathways than in non-metabolic pathways (Fig. 5a). In the tissues dominated by an inflammatory phenotype, such as intestine, liver, kidney, and retina, pathways including glycolysis, carbon, and pyruvate metabolism pathways were upregulated, while oxidative phosphorylation was downregulated (Supplementary Fig. S4), suggesting a link between hypoxia-related energy metabolism and the proinflammatory phenotype of macrophages. Additionally, regulatory transcriptional factors, such as *Cdx2* in the small intestines and *Atf4* in the liver — both are involved in hypoxia pathway — were activated in myopia (Supplementary Fig. S5). In terms of the key regulatory genes, the hypoxia-related *Car1*, aforementioned as upregulated in immune cells, was also the most upregulated gene in the macrophages of myopic mice (Fig. 5b; Supplementary Figs. S6, S7)^24–29^. It was mainly expressed in intestines, liver and kidney (Fig. 5c). Notably, *Car1* was predominantly upregulated among the proinflammatory subsets of macrophages, including Macro_Apoc1 and Macro_S100a (Fig. 5d). These results suggest that the hypoxia pathway may be widely activated in macrophages in myopia.

**Fig. 6:**
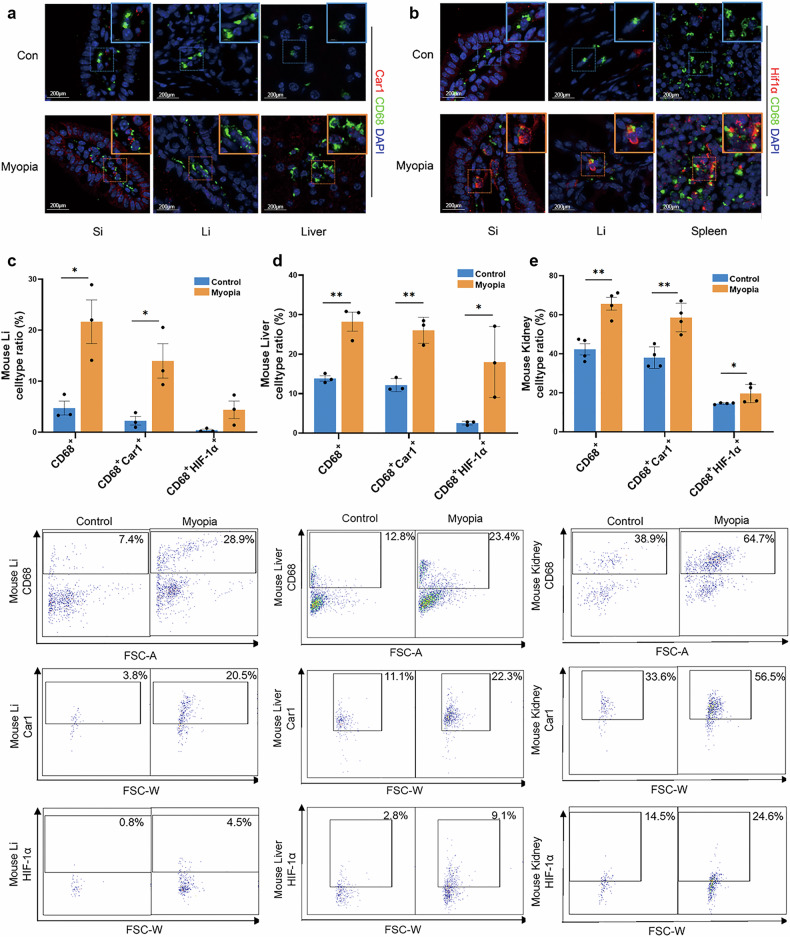
Verification of the activated hypoxia pathway in proinflammatory macrophages across multiple tissues of myopic mice. **a** Immunostaining showing upregulated Car1 in the small intestine, large intestine, and liver of myopic mice compared to controls (*n* = 3). **b** Immunostaining showing upregulated HIF-1α in the small intestine, large intestine and spleen of myopic mice compared to controls (*n* = 3). **c**–**e** Flow-cytometric quantification showed higher ratios of macrophages (CD68^+^), Car1^+^ macrophages, HIF-1α^+^ macrophages in the large intestine, liver, and kidney of the myopic mice compared with those without myopia (*n* = 3 for large intestine and liver; *n* = 4 for kidney). Si small intestine, Li large intestine. **P < 0.01, *P < 0.05.

Furthermore, proinflammatory macrophages and the expression of genes related to the hypoxia pathway in these macrophages in myopia were verified at the protein level (Fig. 6a–e; Supplementary Figs. S8, S9). By immunofluorescence and flow cytometry analyses, we observed a significantly increased ratio of CD68^+^ macrophages and upregulated expression of Car1 in these macrophages within the intestines, liver, and kidney of myopic mice, particularly at 4 weeks (Fig. 6a, c–e; Supplementary Fig. S8). With the progression of myopia, the proinflammatory macrophages and their expression of the hypoxia-responsive gene *Car1* also increased gradually (Supplementary Fig. S8). Moreover, we also identified upregulated expression of the hypoxia-induced factor HIF-1α in the macrophages of the intestines, spleen, liver, and kidney (Fig. 6b–e), along with increased levels of its upstream factor, ROS, which could increase and stabilize HIF-1α, specifically in the macrophages of large intestine and liver of myopic mice (Supplementary Fig. S10). These findings suggest that the activated hypoxia pathway may act as a shared signature in the cross-tissue proinflammatory phenotype of macrophages.

### Inhibition of hypoxia pathway can suppress the proinflammatory phenotype of macrophages across multiple tissues of myopic mice

**Fig. 7:**
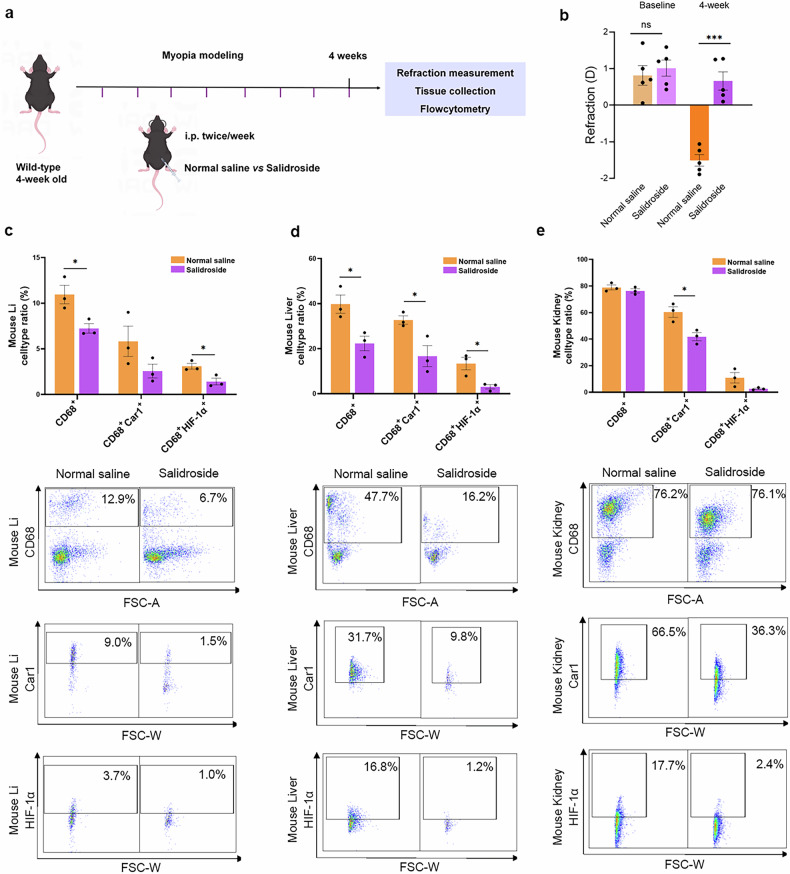
Inhibition of hypoxia pathway can suppress the proinflammatory phenotype of macrophages in multiple tissues. **a** Diagram illustrating the protocol for salidroside injection during myopia modeling in mice. “i.p.” denotes intraperitoneal injection. **b** The refraction at baseline and after 4 weeks of myopia modeling in the normal saline and salidroside groups. Mice injected with salidroside exhibited less myopia compared to those receiving normal saline. **c**, **d** Flow-cytometric quantification showed significantly lower ratios of macrophages (CD68^+^), Car1^+^ macrophages, HIF-1α^+^ macrophages in the large intestine (Li), liver, and kidney of the myopic mice compared with those without myopia (*n* = 3). ***P < 0.001, *P < 0.05; ns no significance.

Importantly, by using salidroside systemically to inhibit the hypoxia pathway in myopic mice (Fig.7a), we found that the ratio of CD68^+^ macrophages and the upregulated expression of Car1 and HIF-1α in these macrophages within the large intestine, liver, and kidney of myopic mice were reduced significantly compared to those injected with normal saline (Fig. 7c–e; Supplementary Fig. S11). Moreover, the refraction was less myopic in the salidroside-treated group than that of the normal saline group (Fig. 7b). The results suggest that the proinflammatory phenotype of macrophages observed across multiple myopic tissues was suppressed by systemic inhibition of the hypoxia pathway, reconfirming the effect of the activated hypoxia pathway in the systemic alterations of proinflammatory macrophages in myopia.

### Confirmation of the shared proinflammatory macrophage signature and its association with extraocular diseases in myopic patients

**Fig. 8:**
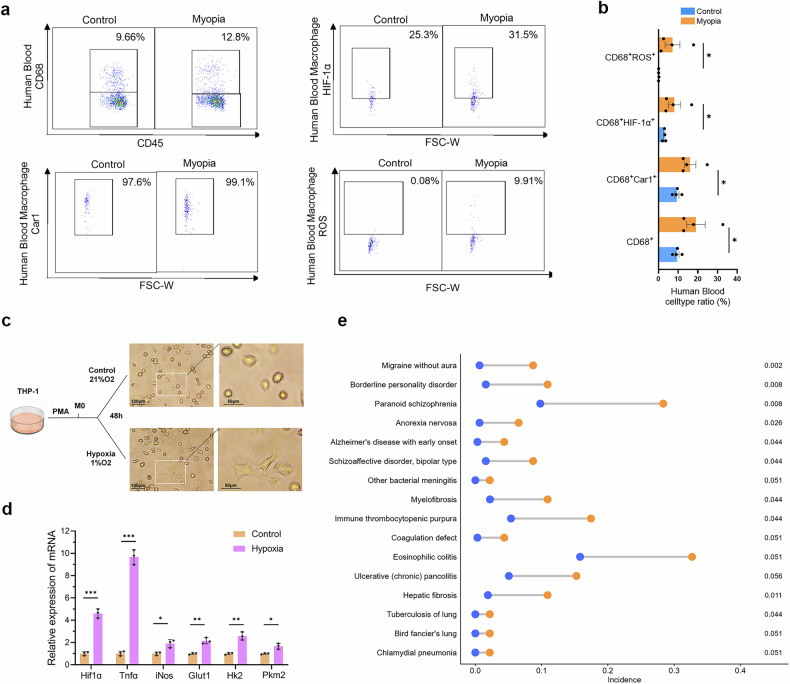
Confirmation of the proinflammatory macrophage signature and its association with extraocular diseases in myopic patients. **a** Flow-cytometric quantification showed higher ratios of macrophages (CD68^+^), Car1^+^ macrophages, HIF-1α^+^ macrophages, and ROS^+^ macrophages in the blood of the myopic patients compared to those without myopia (*n* = 4). **b** Comparison of the ratios of macrophages (CD68^+^), Car1^+^ macrophages, HIF-1α^+^ macrophages, and ROS^+^ macrophages in human blood between emmetropic and myopic patients. *P < 0.05. **c** The diagram illustrating the protocol of human macrophages treated with hypoxia. **d** Human macrophages under hypoxic condition exhibited significantly higher levels of genes related to hypoxia (*Hif-1α*), inflammation (*Tnf-α*, *iNos*), and glycolysis (*Glut1*, *Hk2*, *Pkm2*) as indicated by qPCR (*n* = 3). ***P < 0.001, **P < 0.01, *P < 0.05. **e** Doughnut chart showing the extraocular diseases with a myopia-biased prevalence (myopia-related diseases) and the corresponding prevalence in the myopic patients (orange) and emmetropic patients (blue). Right values indicate adjusted *P* values calculated using the Storey correction.

To further confirm the shared signature of proinflammatory macrophages in humans, we identified an increased ratio of proinflammatory macrophages (CD68^+^), as well as upregulated expression of hypoxia-related *Car1*, *Hif-1α* and ROS, in the blood of myopic patients, compared to those in humans without myopia by flow cytometry (Fig. 8a, b). There was no difference in baseline characteristics including age, sex, or family history between the control and myopic patients (Supplementary Table S1). To further validate the role of the hypoxia pathway in human proinflammatory macrophages, we conducted in vitro experiments. Human macrophages cultured under hypoxic conditions for 48 h demonstrated significantly elevated expression levels of genes associated with hypoxia (*Hif-1α*), inflammation (*Tnf-α*, *iNos*), and glycolysis (*Glut1*, *Hk2*, *Pkm2*) (Fig. 8c, d). These results indicate that hypoxia can enhance the glycolytic process and drive the pro-inflammatory polarization of human macrophages, which is consistent with the findings from animal experiments above.

Despite the shared hypoxia-related signature of proinflammatory macrophages across organs in myopic mice and humans, its clinical relevance remained unknown. To verify the clinical link between myopia and diseases of other organs, based on data from 114,661 participants in the UK Biobank, we compared the incidence rates of 989 disease phenotypes between control and myopic subjects. Totally, 16 extraocular diseases with a myopia-biased prevalence (10 with adjusted *P* < 0.05; 6 with unadjusted *P* < 0.05 and adjusted *P* value close to 0.05) were defined as myopia-related diseases, including Alzheimer’s disease with early onset, myelofibrosis, hepatic fibrosis, etc. (Fig. 8e). Moreover, we also found a close association between myopia and the prevalence of kidney disease (*P* = 0.0209) and allergic rhinitis (*P* = 0.0353) in a cohort of Asian patients.

Evidence suggests that proinflammatory macrophages play a critical role in most of the above myopia-related diseases. In the case of migraines, macrophage infiltration into the nerve tissue contributes to neuroinflammation and sustains neuropathic pain^30^. In individuals with schizophrenia, an increase in perivascular macrophages in the brain enhances inflammation and reduces neurogenesis^31,32^. In the early stage of Alzheimer’s disease, proinflammatory macrophages contribute to synaptic loss and memory deficits^33,34^. Proinflammatory macrophages are also implicated in the pathogenesis of immune diseases^35^. Immune thrombocytopenic purpura is a bleeding disorder characterized by the premature destruction of platelets by macrophages^36^, and myelofibrosis is characterized by a BM-macrophage-mediated inflammatory environment^37^. As to digestive organs, the progression of both ulcerative colitis and liver fibrosis involves the infiltration of proinflammatory macrophages^38–40^.

Overall, we uncover the presence of multi-organ crosstalk in myopic patients, which is potentially associated with a hypoxia-related proinflammatory macrophage signature.

## Discussion

Myopia, a growing public health concern, remains inadequately understood and treated due to its unclear underlying mechanisms. Traditionally viewed as an ocular disease, recent studies have shown inflammation in both the eyes and brains of myopic individuals, suggesting extraocular pathology. However, connections between myopia and extraocular diseases are underexplored. In this study, we used a murine model and scRNA-seq to identify immune changes across multiple organs in myopia. We found a proinflammatory macrophage phenotype, driven by hypoxia pathways, present in both mouse and human myopic tissues. In vitro, hypoxia stress enhanced glycolysis and proinflammatory gene expression in macrophages. The alteration in proinflammatory macrophages and progression of myopia in mice could be suppressed by systemically inhibiting the hypoxia pathway. Additionally, 16 extraocular diseases showed a myopia-associated prevalence in a large cohort, with shared risk genes in hypoxia pathways. These findings point to possible multi-organ crosstalk and highlight the hypoxia pathway in macrophages as a potential therapeutic target for myopia.

Previous research on myopia has predominantly focused on the eye. While in recent years, exploration in cross-organ communication has helped the breakthrough in mechanisms of diseases. Our earlier work revealed that a link between eye and brain may promote the myopia-related anxiety, prompting us to delve deeper into the multi-organ crosstalk in myopia^9,10^. In this study, we identified higher inflammatory response scores in multiple organs using scRNA-seq. Notably, the eye, immune and digestive tissues exhibited an increasing trend in abundance, distinct gene expression and heightened interaction activity in macrophages. Therefore, we speculated that macrophages may play a pivotal role in the multi-organ alterations of the myopic mice.

Importantly, we uncovered a shared proinflammatory phenotype of macrophages across multiple tissues in myopic subjects. The increased proinflammatory phenotype of macrophages in intestines, liver, and kidney was closely associated with the progression of myopia. Previous studies have suggested a link between myopia and the risk of intraocular microinflammation^41^, and proposed that scleral macrophages may contribute to myopia development^8^. In the current study, we observed that the proinflammatory macrophage signature is present across multiple tissues in myopic mice, particularly in the organs with higher enrichment score of inflammatory response gene set. Specifically, the intestines and liver exhibited increased Macro_Apoc1 and Macro_S100a subsets. The former expresses a high level of *CD5L* which inhibits macrophage apoptosis^42^. The latter expresses a high level of *S100a9/S100a8* which promotes glycolytic stimulation and production of proinflammatory cytokines such as IL-1β, TNF-α and CCL2^20,21^. Given that proinflammatory macrophages can migrate across different tissues and adapt to tissue-specific environments, investigating their shared signature may implicate a new therapeutic target for multi-organ control of myopia^43–45^.

The activation of hypoxia pathways may be a potential driver of the pan-tissue proinflammatory phenotype of macrophages in myopia. Among the upregulated DEGs, *Car1* stood out as the most significant and was observed in the organs gaining higher inflammatory response scores in myopia, such as the large intestine, liver, and kidney. Carbonic anhydrases, enzymes involved in hypoxia-induced metabolism, have been implicated in promoting inflammation in various pathologies, including osteoarthritis, cardiovascular disease, infection, and carcinoma^24–27^. Moreover, hypoxia-related pathways, such as HIF-1α signaling, regulate the energy metabolism, particularly the balance between glycolysis and oxidative phosphorylation of macrophages, and promote inflammation^46–48^. Studies have shown that hypoxia-related immunometabolism, encompassing glycolysis, oxidative phosphorylation, and aerobic glycolysis, may serve as the metabolic basis for the reprogramming of myeloid cells^49,50^. Our scRNA-seq data suggested a trend of enhanced glycolytic pathway and reduced oxidative phosphorylation shared by the tissues with higher inflammation levels. The study also showed that hypoxia could enhance the glycolysis in macrophages and promote their proinflammatory signaling in vitro, thus confirming the effect of activated hypoxia pathway on the systemic proinflammatory phenotype of macrophages among myopic subjects.

Intervention targeting the hypoxia pathway may reverse the systemic increase in proinflammatory macrophages and the progression of myopia. Salidroside, a common anti-hypoxic drug, has been reported to suppress the upregulation of HIF-1α and inhibit the progression of experimental myopia in guinea pigs by periocular injection^50^. In this study, salidroside was found to suppress the infiltration of proinflammatory macrophages, their expression of hypoxia-related Car1 and HIF-1α, and further the progression of myopia. Studies also revealed that salidroside could regulate HIF-1α signaling and ameliorate the inflammation mediated by myeloid cells^51,52^. Thus, the hypoxic pathway of macrophages may serve as a potential target for systemic and comprehensive interventions in myopia and its related complications in the future.

More importantly, we identified extraocular diseases related with myopia, and their link to proinflammatory macrophages. Analyzing data from 114,661 participants with 989 disease phenotypes, we found that myopic patients had higher prevalence of 16 extraocular diseases. Interestingly, most of these diseases are similar to myopia, as the proinflammatory macrophage phenotype plays a critical role in their pathogenesis^30–40^. Hypoxia-induced metabolic regulation promotes the proinflammatory function of macrophages, which further contributes to the progression of Alzheimer’s disease, ulcerative colitis, and tuberculosis^34,35,40^. Furthermore, the linkage between systemic proinflammatory macrophages and activation of hypoxia pathway can also be observed in the blood of myopic patients, reconfirming the findings in myopic mice. Other research also indicates a link between myopia and systemic inflammation^41^. Given the correlation between refraction and proinflammatory markers, monitoring myopia progression and systemic inflammatory risks via the levels of specific pro-inflammatory macrophage subsets and their hypoxia-related genes in the blood may act as a potentially impactful avenue. Future research on clinical intentions will be needed.

Previous studies, predominantly considered myopia as a localized disease, focused solely on single-cell analysis of the retina, sclera and lens in myopic eyes. Here, we present a shared signature of macrophages across organs linking myopia and extraocular diseases: during the development of myopia, the eye, immune and digestive tissues shared a proinflammatory phenotype of macrophages, which may be triggered by the activation of hypoxia pathways, thereby potentially modulating the energy metabolic balance between glycolysis and oxidative phosphorylation (Supplementary Fig. S12).

This study has some limitations. Firstly, the samples for single-cell sequencing were derived from a small number of mice, and tissues from the same group were pooled by organ to ensure cost-effectiveness. Yet, we applied a widely used computational method to deconvolve information from pooled samples. Although the efficiency and accuracy of demultiplexing with biological replicates might affect the results, we validated the main findings through in vivo and in vitro experiments, such as immunofluorescence and flow cytometry analyses. Future research incorporating sequencing data from larger human cohorts or integrating multi-omics approaches will be more beneficial for a comprehensive understanding of the systemic mechanism in myopia. Secondly, cell proportion analyses and differential gene expression may be confounded by variability in the number of cells obtained from each tissue under different conditions. However, our focus was on the commonalities among organs, and both in vivo and in vitro experiments verified our findings. Recent spatiotemporal sequencing will facilitate a more comprehensive understanding of the organ-specific characteristics.

In conclusion, our study reveals the presence of organ–organ communication in myopia and emphasizes the role of the hypoxia pathway in proinflammatory macrophages as a shared mechanism across organs in both the murine model and humans. This may pave the way for a more comprehensive and patient-centered approach to addressing this global health challenge.

## Materials and methods

### Ethics

This study was reviewed and approved by the Ethical Committee of the Eye & Ear, Nose, and Throat Hospital of Fudan University, Shanghai, China (No: 2020068). All experiments were performed in accordance with the Declaration of Helsinki and the relevant guidelines and regulations. Animal experiments conformed to the ARVO Statement for the use of animals in research. Written informed consent was obtained from each patient.

### Patients

For exploration of myopia-related diseases, we collected data from 114,661 participants in the UK Biobank database who had mean spherical equivalent (MSE) measurement and extracted 989 disease phenotypes (labeled as Diagnoses-main ICD10) for correlation analysis between myopia and various diseases. The myopia group consisted of patients with MSE less than –6 D, while the emmetropic group had MSE between –0.5 D and 0.5 D. To verify the applicability in Asian population, data from 1723 participants with refraction available in the KNHANES database were also analyzed. For flowcytometry, myopic patients were defined as those with AL ≥ 24.5 mm in both eyes, and the control group was defined as emmetropic patients with a 22–24.5 mm AL in both eyes. Blood samples were collected in 5 mL EDTA-treated tubes and used for isolation of PBMCs with a Ficoll gradient. PBMCs from one individual were used as one sample.

### Identification of myopia-biased diseases

Variance analysis of disease incidence between the emmetropic and myopic groups were performed using R (4.2.1) packages — stats (4.2.1) and qvalue (2.15.0), followed by *P* value adjustment (the Storey correction), which finally defined 16 myopia-related diseases.

### Animals

Four-week-old wild-type mice (C57BL/6 J) were obtained from SLAC Laboratory Animal Co., Ltd. and reared in specific pathogen-free barrier systems under a consistent 12-h light/dark cycle (light on from 7 a.m. to 7 p.m.). The humidity was kept at 40%−60% and the room temperature was kept at 21 °C.

### Defocus-induced myopic mouse model

The refractive states of both eyes of awake mice were initially measured automatically using an automatic eccentric infrared refractometer (Steinbeis Transfer Center, Stuttgart, Germany). Only mice with a binocular refraction difference of ≤ 1 D were considered qualified and included in further experiments. Subsequently, they were assigned to either the sham-treated control group (Con) or the myopia group randomly. In the myopia group, a frame was fixed on the mouse head and –25 D lenses were attached to the frame in front of both eyes. In the control group, only the frame was fixed on the mouse head without attaching any lenses. The modeling period extended over four weeks, during which the mice were maintained in an identical, optimal environment with uniform density, ample access to water and food, and consistent cleanliness^53,54^. The integrity and position of the lenses and frames were checked daily and adjusted if necessary. Prior to sampling, the refractive status of both eyes of each mouse was reassessed using the same method as described above. Additionally, to investigate the alterations with the progression of myopia, refraction status and multiple tissues were collected at 2 weeks, 4 weeks, and 6 weeks during the modeling.

### Tissue dissociation and cell isolation

Anesthesia was induced by intraperitoneal injection of 0.8% pentobarbital sodium before blood collection via retro-orbital bleeding and systemic perfusion with isotonic saline solution. Multiple tissues, including ocular tissues (choroid and retina), brain tissues, immune tissues (blood, bone marrow, spleen, and thymus), and other organs (intestines, liver, lung, kidney, adrenal gland), from 6 mice in each group were collected and dissected. The detailed cell dissociation procedures for the above tissues were provided in the Supplementary Methods.

### scRNA-seq

Multiplexed scRNA-seq was performed for single-cell suspension of multiple tissues, excluding the brain, using cell hashing on the 10x Genomics Chromium Platform. Single cells were transferred to a new 5 mL tube and suspended in 1 mL of pre-chilled PBS with 10% fetal bovine serum. Subsequently, 5 μL of Human TruStain FcX was added, followed by gentle mixing and a 10-min incubation at 4 °C. The pre-mixed Antibody Mix supernatant and FACS antibody pool were then introduced. After 30 min of incubation in darkness at 4 °C, the cells underwent thorough washing. Using the 10x Chromium Controller and Single Cell K Chip (v3.1 chemistry), cells were encapsulated into droplets with gel beads. The scRNA-seq library was constructed using Chromium Single Cell 5’ Gel Beads and Library V2 Kit, following the 10x Genomics protocol. Subsequently, cells were processed in each channel of a Bio-Rad C1000 Touch thermal cycler for reverse transcription and library preparation. The average fragment length of the cDNA library was quantified with a Fragment Analyzer and a Kapa Library Quantification Kit for Illumina. Libraries were sequenced in equimolar pools using the Illumina NovaSeq 6000 S4 Reagent Kit v1.5. Brain tissue samples from six mice were processed for nuclear isolation and scRNA-seq on the 10x Genomics Chromium Platform. Detailed processes were provided in Supplementary Methods.

### Data processing and analysis

The raw sequencing data files underwent initial processing against the reference genome using the 10x Genomics CellRanger software (v5.0.0). Subsequently, the expression matrices were analyzed utilizing the Seurat package (v4.0.4)^55,56^ in the R software environment (v4.1.2). Doublets were identified using the DoubletFinder package (v2.0.3)^57^ and excluded from subsequent analyses. To reduce the influence of cell number variability and increase reliability, strict quality control on cell data, filtering out low-quality cells, and normalization were conducted. Cell counts in different tissues were provided in Supplementary Table S2. Demultiplexing was performed with souporcell^58^.

The unique molecular identifier (UMI) count matrix was further processed using Seurat (v4.0.4). Integration of all cells by sample ID was achieved using Harmony^59^. Cell clustering was conducted based on the top 30 principal components with a resolution of 1 using the FindClusters function. Nonlinear dimensionality reduction techniques such as t-SNE or Uniform Manifold Approximation and Projection (UMAP) were employed to project high-dimensional cellular data into two-dimensional space within Seurat. Cell types were annotated using established marker genes, with the possibility of subsequent sub-clustering. Following the initial cell labeling, manual re-annotation was performed to group cells with similar expression profiles and separate those with distinct patterns, thus enhancing interpretability of intercellular differences. Cell type annotation post-clustering was conducted using SingleR (v1.7.1)^60^, which identifies cell types based on similarity of expression patterns between query and reference cells. Data visualization was accomplished using various tools including Seurat (v4.0.4), ggplot2 (v3.5.0), dittoSeq (v1.5.2), patchwork (v1.2.0), cowplot (v1.1.1), ggrepel (v0.9.3), pheatmap (v1.0.12), ComplexHeatmap (v2.15.1), Mfuzz (v2.54.0)^61^, and ClusterGVis (v0.1.1). Gene Set Enrichment Analysis (GSEA) was performed on DEGs across tissues using the clusterProfiler package (v4.14.6) to identify biologically relevant pathways. The KEGG database served as the reference annotation to identify significant metabolic pathways. Enrichment results were visualized via the ggridges package (v0.5.6) as density ridgeline plots, which effectively illustrate the distribution of enrichment scores across prioritized metabolic pathways. Single-cell rank-based gene set enrichment analysis for “inflammatory response” was performed using the irGSEA package to calculate each cell’s gene score, according to the MsigDB database. Following the identification of major cell types, macrophage lineages were classified based on specific cellular markers. Then, cell–cell interaction analysis^62^ was conducted (Supplementary Methods).

### Experimental hypoxia in macrophages in vitro

THP-1 cells were induced with PMA (100 ng/mL) for 48 h to establish macrophages. These cells were cultured at 37 °C in a humidified incubator with 5% CO_2_ and ~21% O_2_. Subsequently, the cells were seeded at a density of 5 × 10^5^ in 35-mm culture dishes and incubated for 24 h prior to hypoxic treatment. The experimental group was cultivated at 37 °C in a humidified incubator with 1% O_2_, whereas the control group remained under the original culture conditions. After 48 h, the cell status was observed, and the cells were collected for RNA extraction and qPCR. The primers are provided in the Supplementary Table S3.

### Anti-hypoxic drug intervention

Anti-hypoxic drug salidroside was intraperitoneally injected into myopic mice at a dose of 50 mg/kg body weight during the myopia modeling. The injections were performed twice per week, and this treatment was consistently carried out for a total of 4 weeks. The control mice were treated with normal saline at the same dose. At the end of myopia modeling, refraction status was measured for each mouse. For flowcytometry, the intestine, liver and kidney tissues were collected for further experiments.

### Immunofluorescence staining and flow cytometry analysis

Tissues were fixed in 4% formalin for 1 day, embedded in paraffin, and sectioned at 5 µm. Sections were dewaxed in dewaxing solution (G1128, Servicebio), ethanol, and water. Antigen retrieval was performed by microwaving in citrate solution (G1202, Servicebio) at medium heat for 8 min, resting for 5 min, then at medium-low heat for 7 min. After cooling, slides were washed thrice in PBS (G0002, Servicebio), incubated with 3% hydrogen peroxide for 25 min, and washed again. Blocking was done with 3% BSA for 30 min. Primary antibodies were incubated overnight at 4 °C, followed by secondary antibody incubation for 50 min at room temperature. After washing, slides were stained with DAPI for 10 min, treated with an autofluorescence quenching agent (G1221, Servicebio), and rinsed for 20 min before mounting. Images were captured using a fluorescence microscope (NIKON ECLIPSE C1).

Live-Dead staining was performed using FVS506 (65-0866-18, Thermo Fisher Scientific) before cells were incubated with Fc receptor Block (564219 for human, 553142 for mouse, BD Biosciences). Cells were then stained with antibodies in staining buffer (554656, BD Biosciences). Fixation and permeabilization were done using BD Cytofix/Cytoperm Kit (51-2090KZ, BD Biosciences), followed by intracellular staining. Flow cytometry data were acquired using BD FACSCanto II (BD Biosciences) and analyzed with FlowJo (v10, TreeStar).

### Statistics

Data are presented as means ± SEM. Statistical analyses were performed using a two-tailed Student’s *t*-test in PRISM software (GraphPad 6 Software) to compare the differences between the control and myopia groups assuming equal variance. Two-sided *P* < 0.05 is considered statistically significant. *, **, *** and **** indicate *P* < 0.05, *P* < 0.01, *P* < 0.001 and *P* < 0.0001, respectively.

## Supplementary information

Supplemental Information

Supplementary Data S1

## Data Availability

The datasets generated or analyzed in the current study were deposited in public database NODE (https://www.biosino.org/node; access code: OEP00006545) and are available from the corresponding author upon reasonable request. Source data are provided in Supplementary Data S1.
