# Supplementary material for: Single-cell profiling reveals a shared proinflammatory macrophage signature across multiple organs in myopia
Source: Cell Discov. 2025 Dec 2;11:97. doi: 10.1038/s41421-025-00835-8 (PMC12672714; doi:10.1038/s41421-025-00835-8)
Supplement: Supplementary file 1 — Supplemental Information [file 41421_2025_835_MOESM1_ESM.pdf]

Supplementary Information

Single-cell profiling reveals a shared proinflammatory macrophage signature across multiple organs in myopia

Jiaqi Meng[1,2,+], Ye Zhang[1,2,+], Mengchao Zhu[1,2,+], Yu Du[1,2], Yunqian Yao[1,2], Shuyu Liu[1,2], Wenwen He[1,2], Xiangjia Zhu[1,2,3*#]

[1]Department of Ophthalmology, Eye & ENT Hospital, Fudan University, Shanghai, China.

[2]Key laboratory of Myopia and Related Eye Diseases, NHC; Key laboratory of Myopia and Related Eye Diseases, Chinese Academy of Medical Sciences, Shanghai, China.

[3]Key Laboratory of Medical Neurobiology, Fudan University, Shanghai, China

[*] These authors contributed equally to this work

[+] Correspondence: Xiangjia Zhu, PhD, MD. Eye & ENT Hospital, Fudan University, Shanghai 200031, China. E-mail: zhuxiangjia1982@126.com

Includes files: Supplementary Fig. S1-12, Supplementary Table S1, Supplementary Table S2, Supplementary Table S3, Supplementary Methods

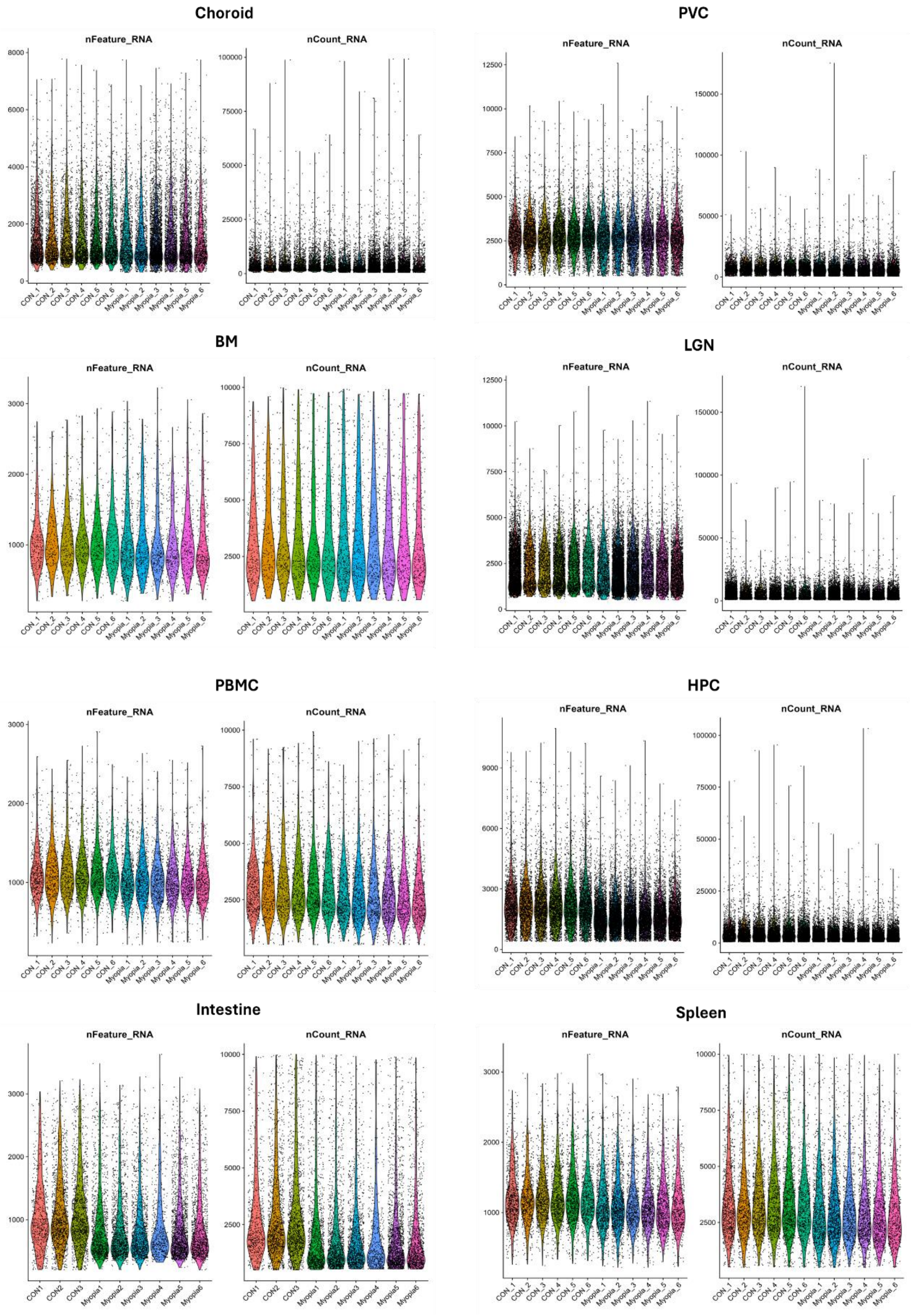

Supplementary Fig. S1. Distribution of gene types and quantities in each sample across multiple organs. Violin plots of the distribution of gene types (nFeature) and quantities (nCount) in each sample among different organs. Con, control; M, myopia; PVC, primary visual cortex; LGN, lateral geniculate nucleus; HPC, hippocampus; PBMC, peripheral blood mononuclear cell; BM, bone marrow.

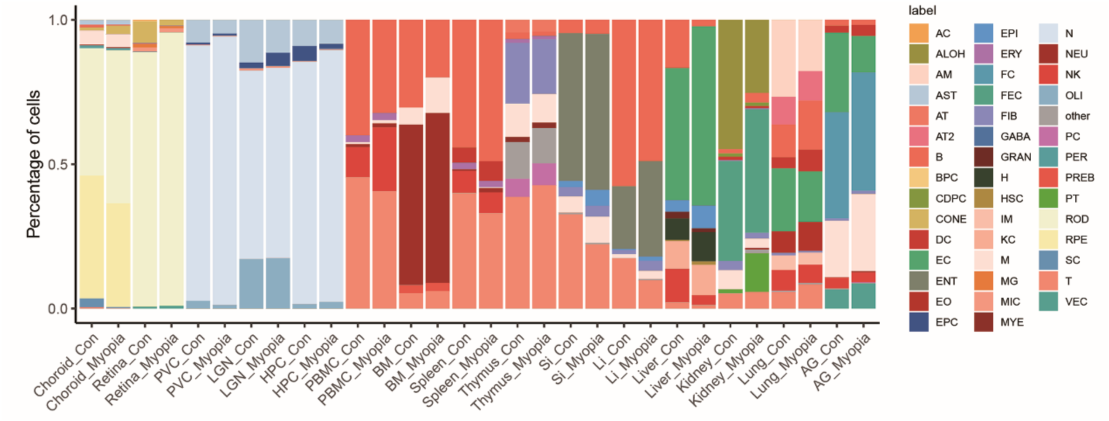

Supplementary Fig. S2. Percentages of different cell types across multiple organs between the control and myopic mice by scRNA-seq. Percentages of all cell types in each tissue betweeen the control (Con) and myopic mice. PVC, primary visual cortex; LGN, lateral geniculate nucleus; HPC, hippocampus; PBMC, peripheral blood mononuclear cell; BM, bone marrow; Si, small intestine; Li, large intestine; AG, adrenal gland. M, macrophage; AM, alveolar macrophage; IM, interstitial macrophage; KC, Kupffer cell; MIC, microglia; T, T cell; AT, activated T cell; B, B cell; PREB, pre B cell; NK, natural killer cell; DC, dendritic cell; EO, eosinophil; NEU, neutrophil; MYE, myeloid cell; GRAN, granulocyte; RPE, retinal pigment epithelial cell; ROD, rod cell; BPC, bipolar cell; CONE, cone cell; AC, amacrine cell; MG, muller glia cell; N, neuron; AST, astrocyte; OLI, oligodendrocyte; SC, Schwann cell, GABA, GABAergic neuron; EPC, ependymal cell; AT2, AT2 cell; ENT, enterocyte; H, hepatocyte; HSC, hepatic stellate cell; ALON, ascending loop of henle; CDPC, collecting duct principal cell; PT, proximal tubule cell; EC, endothelial cell; FEC, fenestrated endothelial cell; VEC, vascular endothelial cell; PER, pericyte; FC, fasciculata cell; EPI, epithelial cell; FIB, fibroblast; ERY, erythroid cell; PC, proliferating cell.

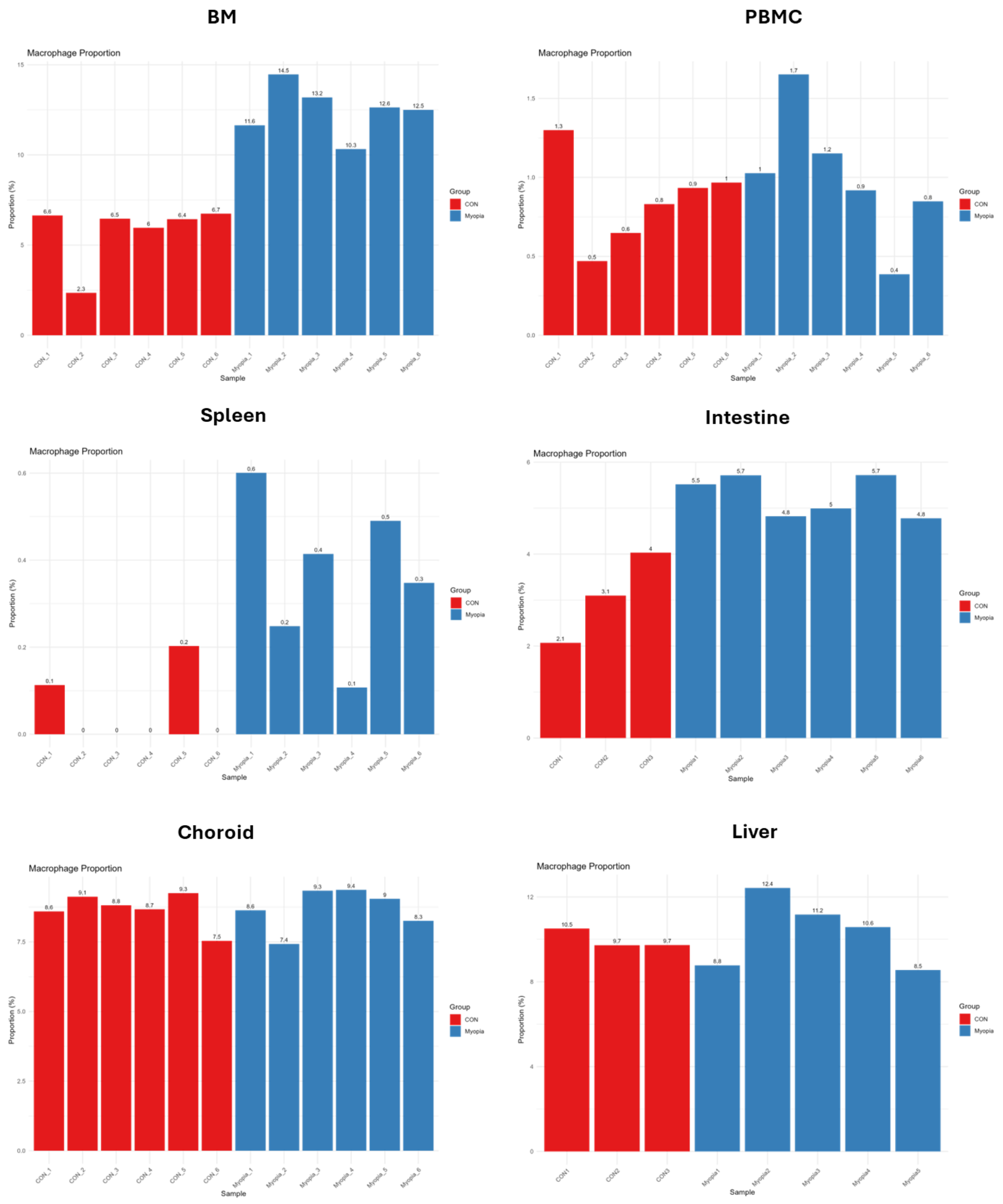

Supplementary Fig. S3. Proportions of macrophages in each control or myopia sample across multiple organs. Proportions of macrophages in each sample among different organs. Con, control; M, myopia; PBMC, peripheral blood mononuclear cell; BM, bone marrow.

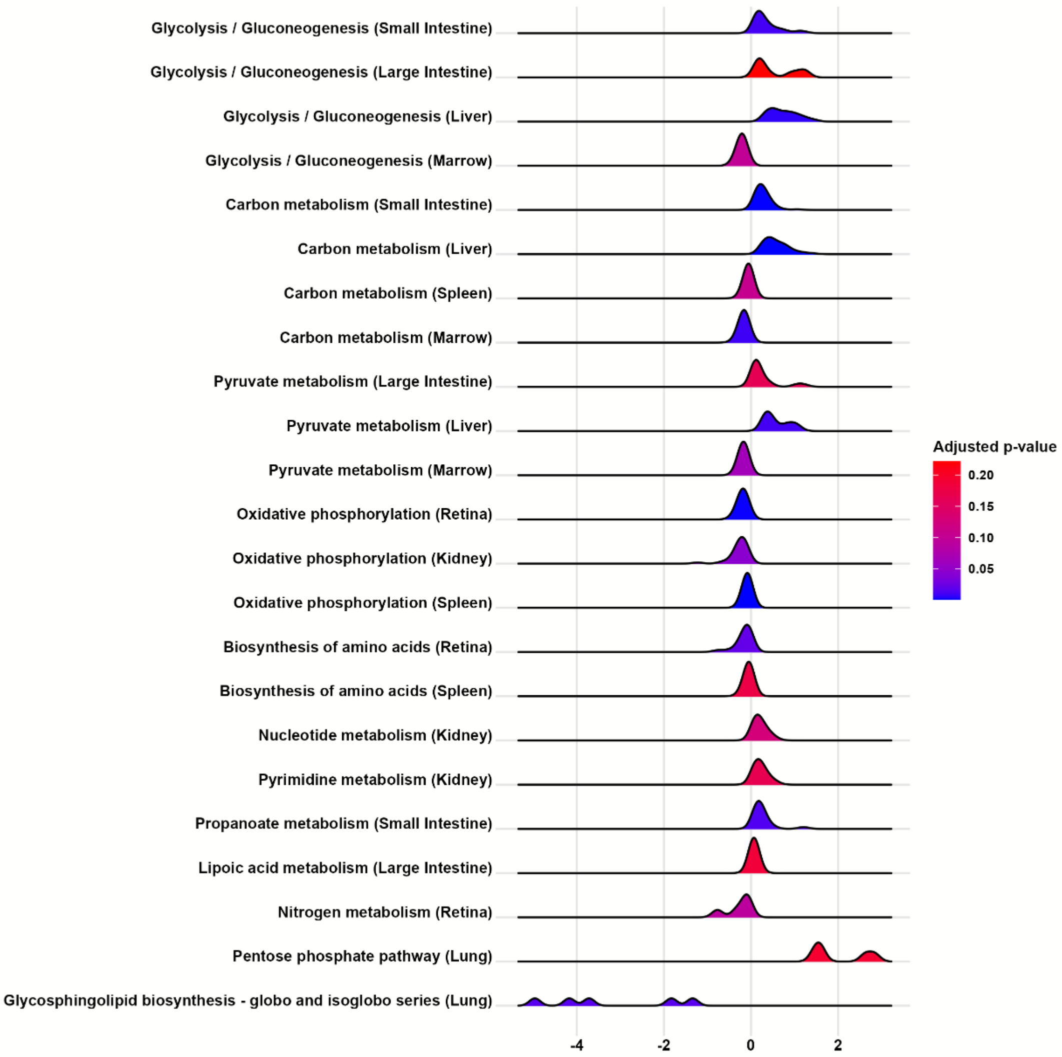

Supplementary Fig. S4. Enrichment pathways in energy metabolism across different tissues of myopic mice. In the tissues dominated by inflammatory phenotype, such as intestine, liver, kidney, and retina, pathways including glycolysis, carbon, and pyruvate metabolism pathways were upregulated, while oxidative phosphorylation was downregulated. The glycolysis, carbon and pyruvate metabolism pathways in bone marrow were downregulated. Values over zero indicate upregulation, while those less than zero indicate downregulation.

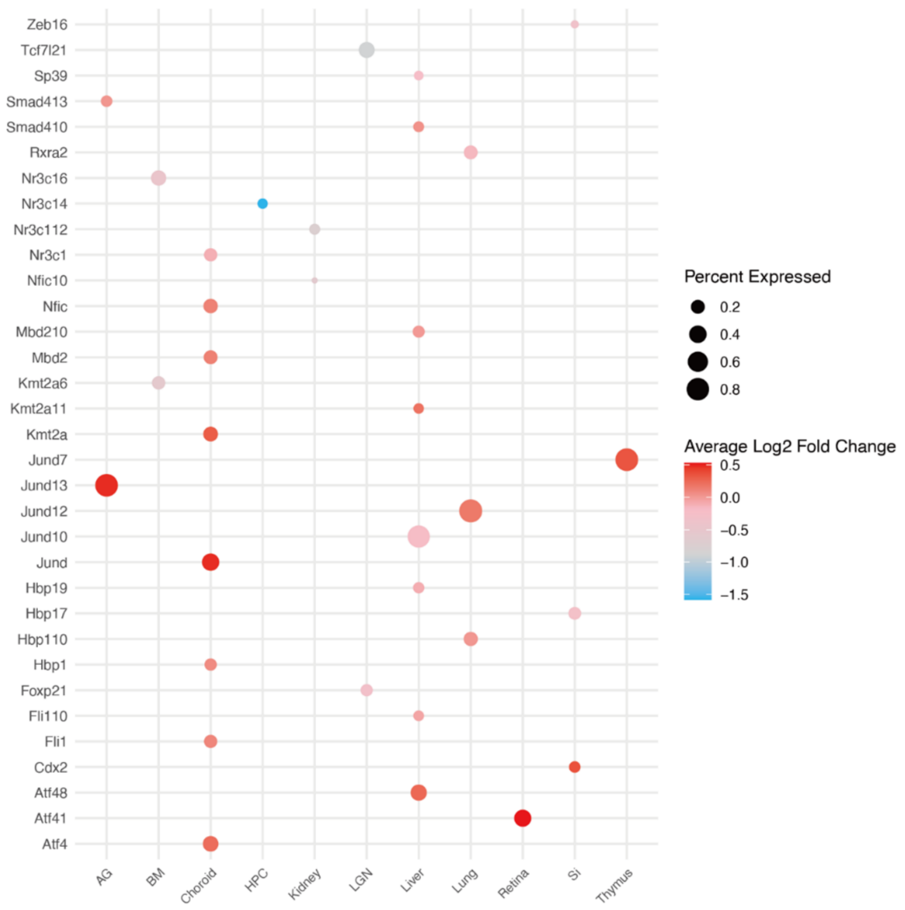

Supplementary Fig. S5. Regulatory transcriptional factors in macrophages of myopic mice. The bubble plot showing the differential transcriptional factors (TFs) in macrophages across tissues in the myopia group.

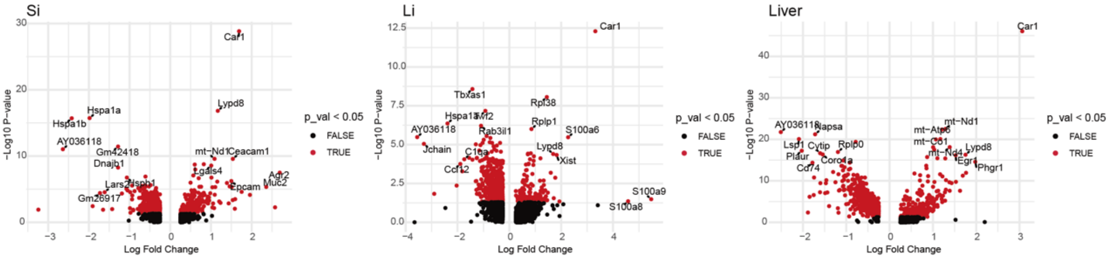

Supplementary Fig. S6. Representative differentially expressed genes (DEGs) of macrophages in intestines and liver tissues of myopic mice. Volcano plots showing the macrophage DEGs in the small intestines (Si), large intestines (Li), and liver of myopic mice. *Car1* upregulation was shared among intestines and liver.

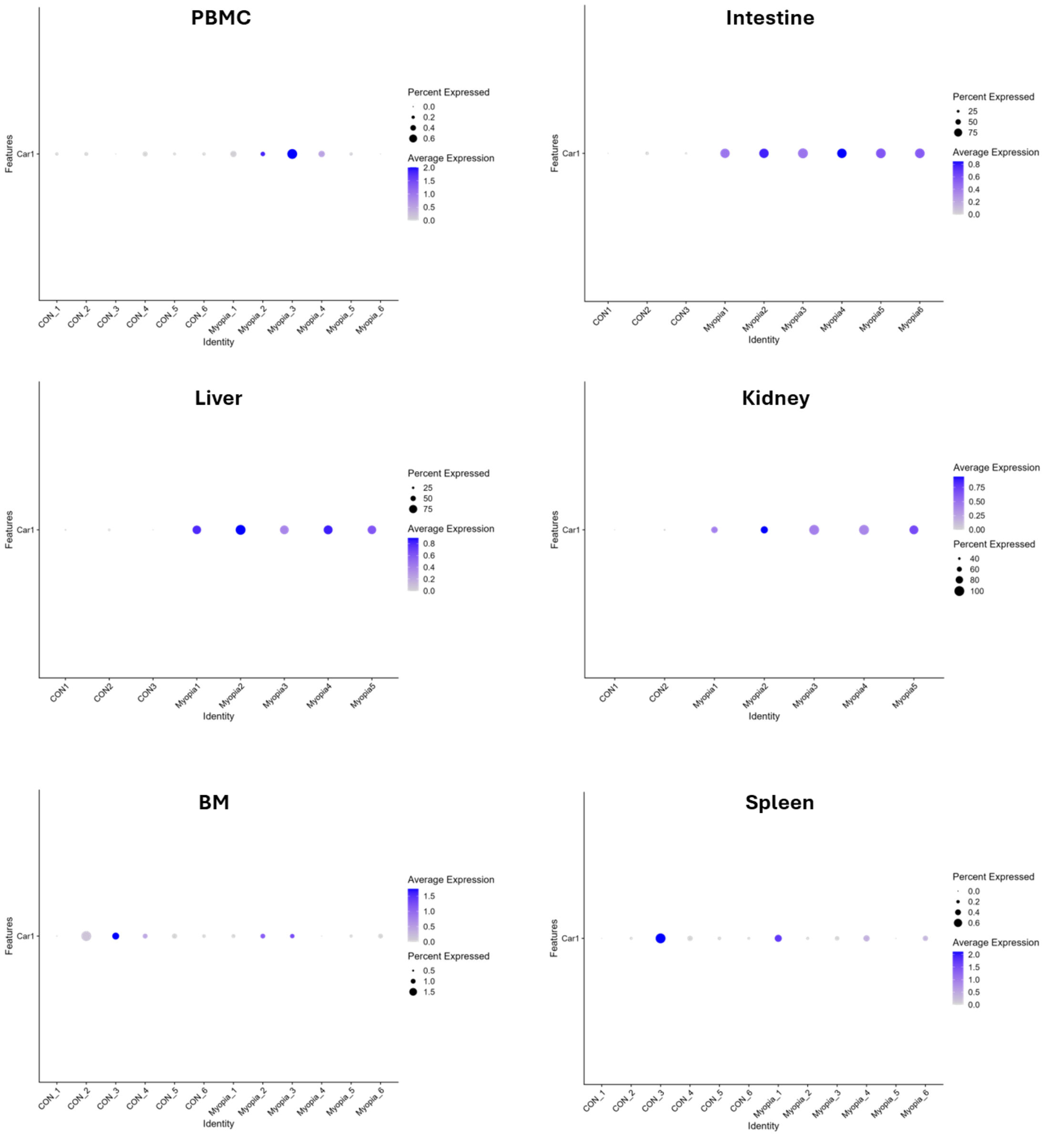

Supplementary Fig. S7. Expression of Car1 in each control or myopia sample across different tissues. The bubble plots showing the Car1 expression levels in the macrophages in each sample across different tissues. Con, control; M, myopia; PBMC, peripheral blood mononuclear cell; BM, bone marrow.

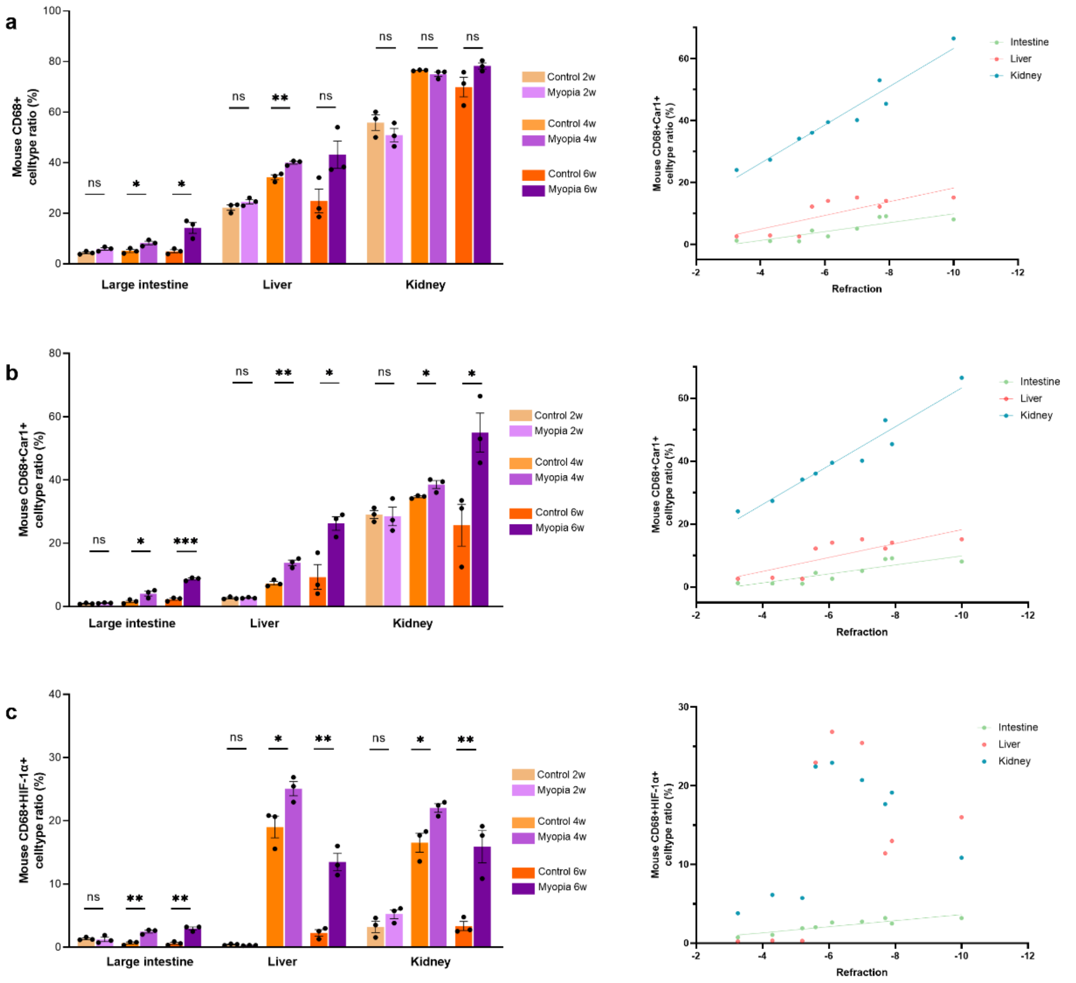

Supplementary Fig. S8. Changes in proinflammatory macrophages and their gene expression related to hypoxia pathway across multiple tissues in myopic mice. Left column shows the ratios of $CD68^+$ macrophages (a), $CD68^+Car1^+$ (b), and $CD68^+HIF\text{-}1\alpha^+$ macrophages (c) in large intestine, liver and kidney at 2-week, 4-week, and 6-week timepoints during myopia modeling (n=3). Right column shows the correlation between myopic refraction and the ratios of macrophage subtypes in intestine, liver and kidney. Solid lines indicate statistical significance. (n=9). *** P<0.001, ** P<0.01, * P<0.05.

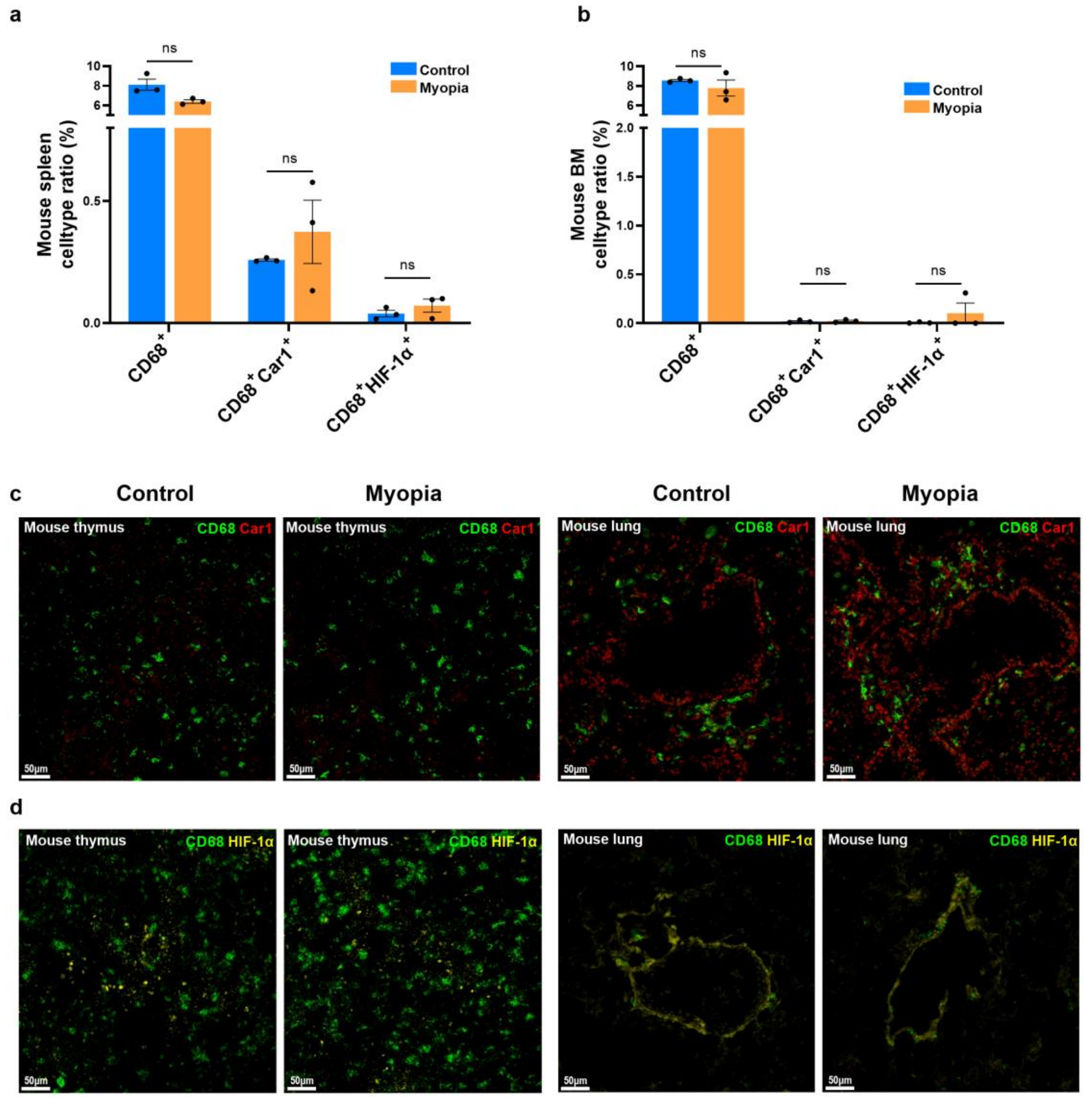

Supplementary Fig. S9. The levels of proinflammatory macrophages and their gene expression related to hypoxia pathway in the spleen, bone marrow, thymus and lung of the myopic mice. (a) Flow-cytometry analysis showed there were no significant difference in the ratios of $CD68^+$ macrophages in the spleen between the control and myopia groups. The ratios of $CD68^+Car1^+$ and $CD68^+HIF\text{-}1\alpha^+$ macrophages showed a slight increase though without statistically significant difference. (*n*=3). (b) Flow-cytometry analysis showed there were no difference in the ratios of $CD68^+$ macrophages and expression of Car1 in the bone marrow (BM) between the control and myopia groups. (*n*=3). (c) Immunostaining showing the expression levels of CD68 and Car1 were not upregulated in the thymus or lung of the myopic mice compared to the controls. Scale bar: 50μm. (d) Immunostaining showing the expression levels of CD68 and HIF-1α were not upregulated in the thymus or lung of the myopic mice compared to the controls. Scale bar: 50μm.

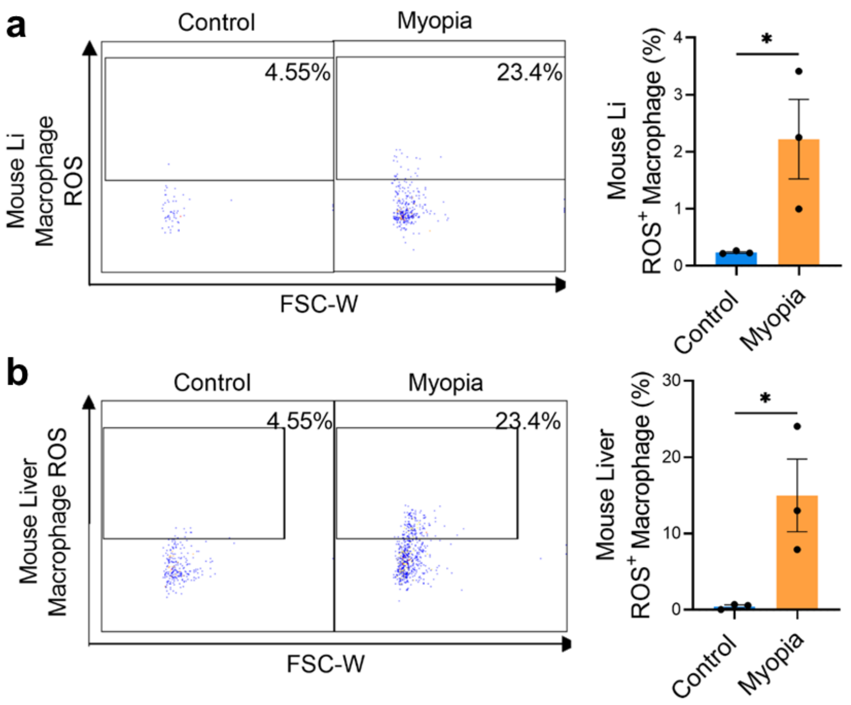

Supplementary Fig. S10. Increased ratios of ROS⁺ macrophages in the large intestine and liver of myopic mice. (a) Flow-cytometric quantification showed higher ratios of ROS^+ macrophages in the large intestine (Li) of myopic mice than those without myopia. (n=3). (b) Flow-cytometric quantification showed higher ratios of ROS^+ macrophages in the liver of myopic mice than those without myopia. (n=3). * P<0.05.

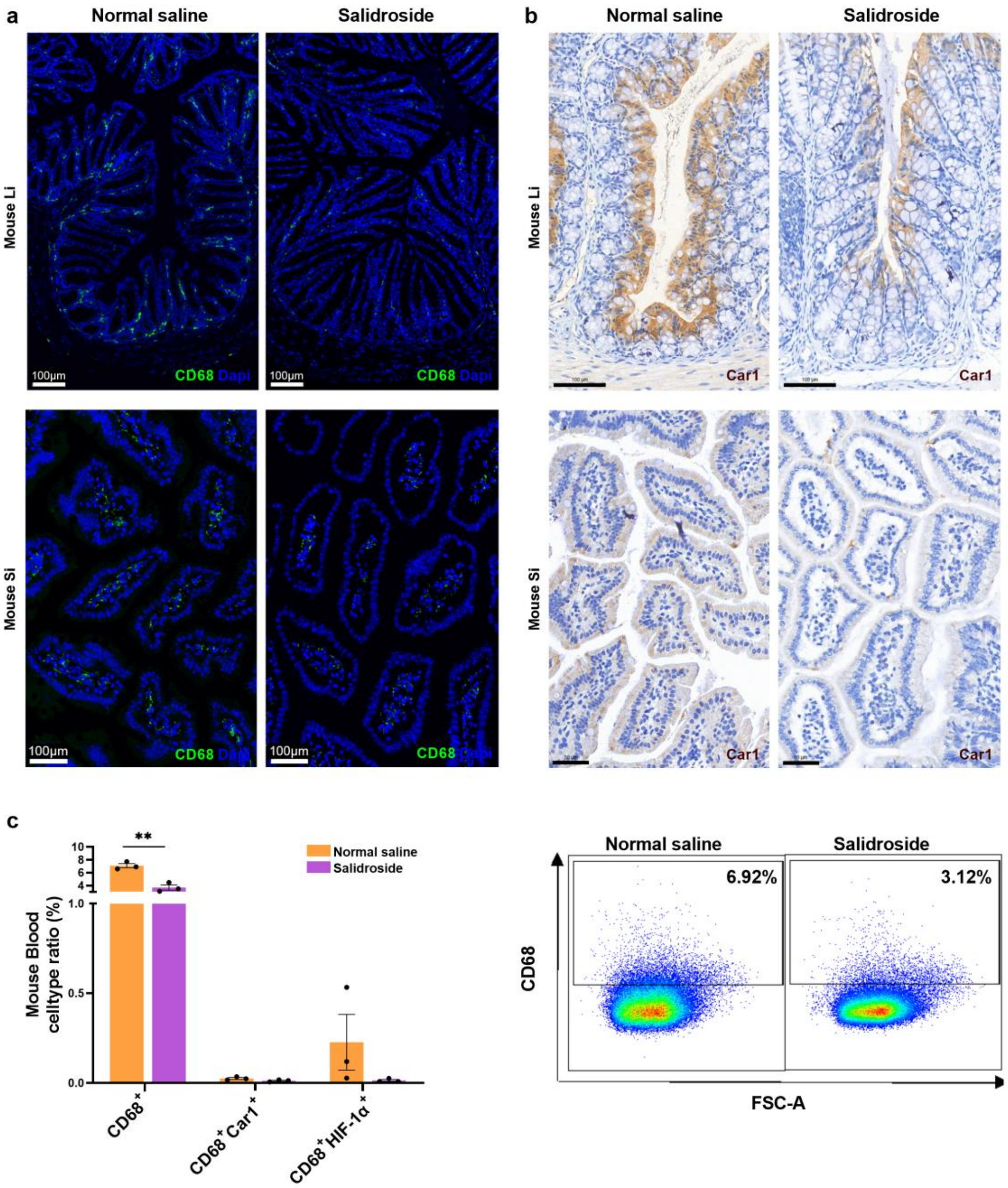

Supplementary Fig S11. Effect of anti-hypoxia drug on the proinflammatory macrophages in the intestines and blood of myopic mice. (a) Immunostaining showing increased CD68-positive macrophages in the large intestine (Li) and small intestine (Si) of the salidroside group compared to those of the normal saline group. Scale bar: 100μm. (b) Immunohistochemistry showing upregulated Car1 in the large intestine (Li, Scale bar: 100μm) and small intestine (Si, Scale bar: 50μm) of the salidroside group compared to those of the normal saline group. (c) Flow-cytometric quantification showed lower ratio of $CD68^{+}$ macrophages in the blood of myopic mice

receiving anti-hypoxia drug salidroside than those receiving normal saline. (*n*=3). ** P<0.01.

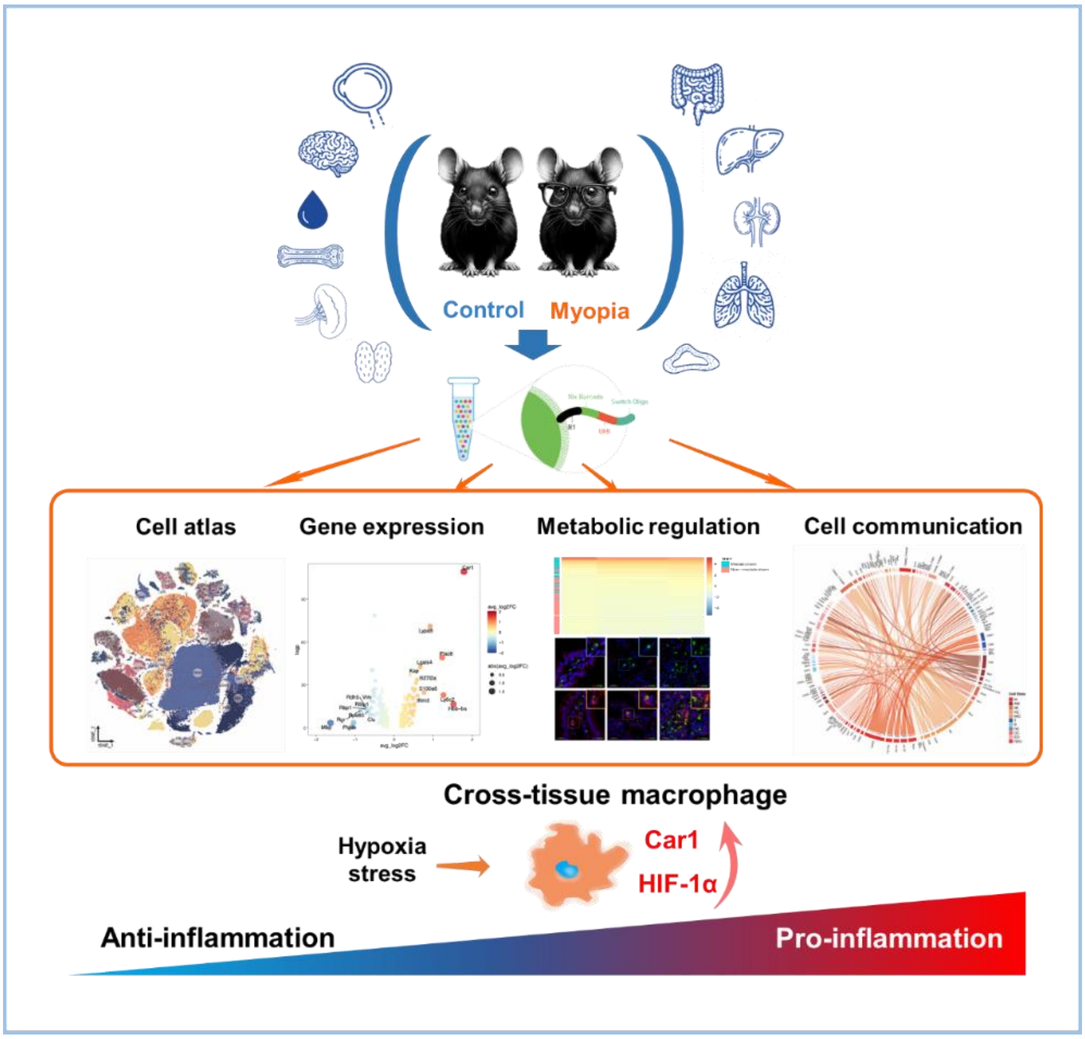

Supplementary Fig S12. Schematic graph for the role of proinflammatory macrophages in the systemic immune remodeling by myopia. Cross-tissue proinflammatory macrophages upregulate Car1 and activate HIF-1α signaling, etc, thereby modulating the hypoxia-related metabolic balance between glycolysis and oxidative phosphorylation, etc, ultimately leading to a systemic immune remodeling by myopia.

Supplementary Table S1. Baseline features of patients for blood sample collection

	Control	Myopia	P value
Age, years	25.3±3.9	25.3±3.9	0.793
Sex, female/male	2/2	2/2	0.757
Refraction, diopter	0.06±0.12	-8.30±1.45	<0.001
Axial length, mm	23.79±0.42	28.08±0.68	<0.001
Family history, present/absent	0/4	0/4	/

Supplementary Table S2. Cell counts of different tissues in scRNA-seq analysis

Cell counts	Control	Myopia
Choroid	16658	14742
Retina	23585	21220
PVC	7092	7824
LGN	10077	13229
HPC	10236	19252
Blood	3134	3595
Bone marrow	1589	2205
Spleen	4818	5399
Thymus	2888	1999
Small intestine	1535	2817
Large intestine	1867	4505
Liver	2033	1900
Kidney	346	1337
Lung	3724	2915
Adrenal gland	1499	1235

Supplementary Table S3. Primer information

h-TNFα-F	CCTCTCTCTAATCAGCCCTCTG
h-TNFα-R	GAGGACCTGGGAGTAGATGAG
h-iNOS-F	TCATCCGCTATGCTGGCTAC
h-iNOS-R	CCCGAAACCACTCGTATTTGG
h-HIF1α-F	GAACGTCGAAAAGAAAAGTCTCG
h-HIF1α-R	CCTTATCAAGATGCGAACTCACA
h- Glut1-F	ATTGGCTCCGGTATCGTCAAC
h-Glut1-R	GCTCAGATAGGACATCCAGGGTA
h-Hk2-F	TTGACCAGGAGATTGACATGGG
h-Hk2-R	CAACCGCATCAGGACCTCA
h-Pkm-F	AAGGGTGTGAACCTTCCTGG
h-Pkm-R	GCTCGACCCCAAACTTCAGA

Supplementary Methods

Cell dissociation

Tissues were promptly sectioned into approximately 1 mm diameter fragments in a chilled environment using pre-cooled DPBS (GIBCO) and placed into a 15 ml centrifuge tube. Two successive washes with pre-cooled DPBS were performed to remove residual debris and non-cellular components.

a. Choroid: Choroid tissues were incubated in a 37°C water bath shaker for 1.5 hours using a Lamina Propria Dissociation Kit (130-097-410 Miltenyi Biotec), papain, 1 mg/ml collagenase IV, and 3 μg/ml DNAse I.

b. Retina: Retina tissues were incubated in a 37°C water bath shaker for 30 minutes with papain.

c. Spleen and thymus: Spleen and thymus tissues were incubated in a 37°C water bath shaker for 30 minutes using a Lamina Propria Dissociation Kit and collagenase I.

d. Intestines: Small and large intestines tissues were incubated in a 37°C water bath shaker for 20-30 minutes using a Lamina Propria Dissociation Kit and collagenase IV.

e. Liver: Liver tissues were treated with collagenase IV (2 mg/mL, 17104019, GIBCO) in a gentleMACS C tube and dissociated with the gentleMACS Dissociator for 45 minutes.

f. Kidney: Kidney tissues were incubated in a 37°C water bath shaker for 30 minutes using a Lamina Propria Dissociation Kit, collagenase IV, and trypsin.

g. Lung and adrenal gland: Lung and adrenal gland tissues were dissociated in a gentleMACS C tube with the Lamina Propria Dissociation Kit using the gentleMACS Dissociator for 45 minutes.

h. Brain: After perfusion, specific brain regions (PVC, LGN, HPC) were dissected from the brain and promptly flash-frozen in liquid nitrogen for subsequent nuclear extraction and sequencing analysis.

The digestion of the aforementioned tissues (excluding the brain) was terminated by adding pre-chilled high-glucose DMEM (GIBCO) supplemented with 10% fetal bovine serum (FBS, GIBCO) to the digested suspension. Subsequently, the cell suspension underwent centrifugation at 1000 rpm at 4°C for 5 minutes, followed by resuspension in Red Blood Cell (RBC) Lysis Buffer (BD Biosciences) at room temperature for 5 minutes, and a further centrifugation at 37°C for 5-20 minutes. This cycle was repeated 2-3 times as necessary until complete removal of the RBC. For the elimination of impurities and multicellular aggregates, the cells were resuspended in 3 ml of pre-

chilled DPBS, filtered through a 70 μm MACS® SmartStrainer (130-098-462, Miltenyi Biotec, Germany), and rinsed twice with DPBS. To eliminate cell debris, the cell suspension underwent sorting using FACS (BD-influin) and was then resuspended in pre-chilled DMEM containing 10% FBS.

Live/dead cell staining with AO/PI was performed, and cell viability was assessed under a microscope to ensure that it exceeded 80%. Following a 5-minute incubation with a tag, the samples underwent two washes with pre-chilled DPBS and centrifugation at 1000 rpm at 4°C. The resulting pellet was resuspended in DPBS containing 0.04% bovine serum albumin (BSA) to achieve a single-cell suspension, which was subsequently utilized for 10X genomic sequencing.

The procedures for cell dissociation of PBMC and BM were conducted as follows:

a. PBMC: Blood samples collected in EDTA-coated tubes were centrifuged at 1600g for 10 minutes at room temperature. Following centrifugation, the upper plasma layer was carefully aspirated, leaving the pellet undisturbed. An equal volume of DPBS at room temperature was then gently added to the pellet and mixed by inversion. The diluted blood was subsequently layered onto an equal volume of Lymphoprep™ (1858, Serumwerk Bernburg AG, Germany) and centrifuged at 500g for 20 minutes at 20°C. The resulting white membrane layer was aspirated, suspended in PBC Lysis Buffer (BD Biosciences), and incubated for 5 minutes at room temperature. Subsequently, the cells were washed twice with DPBS at room temperature.

b. BM: Mouse femurs were flushed with pre-chilled DPBS using a syringe to collect bone marrow tissue. The collected bone marrow suspension was filtered through 70mm MACS® SmartStrainers (130-098-462, Miltenyi Biotec, Germany) and centrifuged at 300g for 5 minutes at 4°C. The resulting pellet was then resuspended in 2 ml of RBC Lysis Buffer (BD Biosciences) and incubated at room temperature for 10 minutes. After incubation, the cells were washed twice with pre-chilled DPBS.

Sc-RNA sequencing for the brain tissues

Brain tissue samples from six mice were processed for nuclear isolation and scRNA-seq on the 10x Genomics Chromium Platform. Approximately 100 mg of brain tissue was homogenized using a cryogenic multi-sample tissue grinder (20xLoose, 40xTight) in 1 mL of pre-cooled lysis buffer containing 0.32M sucrose, 5 mM CaCl2, 3 mM magnesium acetate, 0.1 mM EDTA, 10 mM Tris-HCl pH 8, 1 mM DTT, 0.1% Triton X-100, 1X protease inhibitor, and 0.4 U/mL RNase. The homogenate was then filtered through a 40 μm cell strainer, layered on top of a 1.8M Sucrose Cushion Buffer, and

centrifuged at 2500g for 5 minutes at 4°C. The resulting pellet was washed in 1 mL lysis buffer to remove residual sucrose, followed by resuspension in 1 mL PBS containing 1% BSA and centrifugation at 500g for 5 minutes at 4°C. Finally, the cells were resuspended in PBS with 0.5% BSA. Single-cell suspensions for the 10x Chromium Controller (10x Genomics, Pleasanton, CA) and Single Cell G Chip (v3.1 chemistry, PN-1000120) were prepared, loading approximately 20,000 cells per channel. Single-cell GEMs were generated using the Chromium Controller, and libraries were constructed using the 10x Chromium Single Cell 3' Reagent Kits (v3.1 chemistry PN-1000268). Sequencing was performed using the Illumina NovaSeq 6000 S4 Reagent Kit v1.5, following the standardized 10x capture and library preparation protocols established by 10x Genomics.

Macrophage Subtyping

Clusters 0, 2, 15, and 21 expressing Chil3, CD44, Cxcl2, Lpl, and Ear2 were designated as *Macro Chil3* cells. Clusters 1 and 5, expressing CD74 and histocompatibility 2-related genes, were termed *Macro CD74* cells. Cluster 6, expressing Cd209a, was labeled as *Macro Cd209a* cells. Clusters 3, 4, 29, and 32, expressing mitochondrial-related genes, were denoted as *Macro mt* cells. Cluster 14, expressing Hexb and C1q, was termed *Macro Hexb+C1q* cells. Cluster 26, expressing Hexb and Egr1, was identified as *Macro Hexb+Egr1* cells. Clusters 18, 30, and 33, expressing Meg3, were designated as *Macro Meg3* cells. Clusters 13, 20, 22, and 35, expressing PF4, were termed *Macro PF4* cells. Cluster 7, expressing Cd3, was labeled as *Macro Cd3* cells. Clusters 10 and 11, expressing Sag, were defined as *Macro Sag* cells. Cluster 24, expressing Glul, was identified as *Macro Glul* cells. Cluster 17, expressing Stmn1, was termed *Macro Stmn1* cells. Cluster 31, expressing Star, was designated as *Macro Star* cells. Cluster 8, expressing Ly6c1, was labeled as *Macro Ly6c1* cells. Cluster 12, expressing Apoc1, was designated as *Macro Apoc1* cells. Clusters 25 and 28, expressing S100a, were termed *Macro S100a* cells. Clusters 9 and 23, expressing Ifitm3/6, were designated as *Macro Ifitm3/6* cells. Cluster 34, expressing Ccr7, was labeled as *Macro Ccr7* cells. Cluster 16, expressing Bst2 and Siglec H, was designated as *Macro Bst2-siglech* cells. Cluster 19, expressing Mzb1, was termed *Macro Mzb1* cells. Cluster 27, expressing Ptgds, was labeled as *Macro Ptgds* cells.

Cell–cell interaction analysis

CellPhoneDB V2.0 was employed to elucidate ligand-receptor interactions among single cells, and the findings were visualized using pheatmap (V1.0.12). Considering

the spatial segregation of cells across various tissues, CellChat (V2.1.2) was utilized to analyze ligand-receptor interactions derived from single-cell gene expression matrices originating from diverse tissues. Our analysis focused on receptors and ligands expressed above a user-defined threshold within specific cell clusters. The expression abundance of these ligand-receptor pairs served as a metric for preliminary assessment of cellular communication. To identify biological associations, CellPhoneDB conducted pairwise comparisons across all cell types within the dataset, specifically targeting ligand-receptor pairs significantly enriched in each cell pair.

Antibodies

For immunofluorescence staining: CA1 (DF3885, 1:100), HIF1α (AF1009, 1:100), Anti-CD68 (GB113109, 1:2000), HRP Goat Anti-Rabbit IgG (GB23303, 1:500), CY3 Goat Anti-Rabbit IgG (GB21303, 1:300), DAPI (G1012).

For flowcytometry: anti-CD45 (25-0451-82 for human, 25-0459-42 for mouse, Thermo Fisher) and ROS (16051, AAT Bioquest), anti-CD68 (564943 for human, BD Pharmingen; 404-0681-82 for mouse, Thermo Fisher), anti-HIF-1α (17-7528-82, Thermo Fisher), anti-Car1 (PA5-78896, Thermo Fisher), and secondary antibody (12-4739-81, Thermo Fisher).
